# Mitochondria DNA copy number, mitochondria DNA total somatic deletions, Complex I activity, synapse number, and synaptic mitochondria number are altered in schizophrenia and bipolar disorder

**DOI:** 10.1038/s41398-022-02127-1

**Published:** 2022-08-30

**Authors:** Sujan C. Das, Brooke E. Hjelm, Brandi L. Rollins, Adolfo Sequeira, Ling Morgan, Audrey A. Omidsalar, Alan F. Schatzberg, Jack D. Barchas, Francis S. Lee, Richard M. Myers, Stanley J. Watson, Huda Akil, William E. Bunney, Marquis P. Vawter

**Affiliations:** 1 Functional Genomics Laboratory, Department of Psychiatry & Human Behavior, University of California, Irvine, CA USA; 2 Department of Translational Genomics, Keck School of Medicine, University of Southern California, Health Sciences Campus, Los Angeles, CA USA; 3 Department of Psychiatry and Behavioral Sciences, Stanford University, Stanford, CA USA; 4 Department of Psychiatry, Weill Cornell Medical College, Ithaca, NJ USA; 5 HudsonAlpha Institute for Biotechnology, Huntsville, AL 35806 USA; 6 The Michigan Neuroscience Institute, University of Michigan, Ann Arbor, MI USA; 7 Department of Psychiatry & Human Behavior, University of California, Irvine, CA USA

**Keywords:** Schizophrenia, Bipolar disorder

## Abstract

Mitochondrial dysfunction is a neurobiological phenomenon implicated in the pathophysiology of schizophrenia and bipolar disorder that can synergistically affect synaptic neurotransmission. We hypothesized that schizophrenia and bipolar disorder share molecular alterations at the mitochondrial and synaptic levels. Mitochondria DNA (mtDNA) copy number (CN), mtDNA common deletion (CD), mtDNA total deletion, complex I activity, synapse number, and synaptic mitochondria number were studied in the postmortem human dorsolateral prefrontal cortex (DLPFC), superior temporal gyrus (STG), primary visual cortex (V1), and nucleus accumbens (NAc) of controls (CON), and subjects with schizophrenia (SZ), and bipolar disorder (BD). The results showed (i) the mtDNA CN is significantly higher in DLPFC of both SZ and BD, decreased in the STG of BD, and unaltered in V1 and NAc of both SZ and BD; (ii) the mtDNA CD is significantly higher in DLPFC of BD while unaltered in STG, V1, and NAc of both SZ and BD; (iii) The total deletion burden is significantly higher in DLPFC in both SZ and BD while unaltered in STG, V1, and NAc of SZ and BD; (iv) Complex I activity is significantly lower in DLPFC of both SZ and BD, which is driven by the presence of medications, with no alteration in STG, V1, and NAc. In addition, complex I protein concentration, by ELISA, was decreased across three cortical regions of SZ and BD subjects; (v) The number of synapses is decreased in DLPFC of both SZ and BD, while the synaptic mitochondria number was significantly lower in female SZ and female BD compared to female controls. Overall, these findings will pave the way to understand better the pathophysiology of schizophrenia and bipolar disorder for therapeutic interventions.

## Introduction

Mitochondria are the primary energy-producers of the neural cells as they generate ~90% of neural cell energy in the form of adenosine triphosphate (ATP). Energy production of mitochondria in the brain is critical, as the brain uses over 20% of the energy produced in the body, yet it is only about 2% of adult body weight [1]. Mitochondria generate ATP through a complex set of enzymes called the electron transport chain (ETC). The ETC consists of five protein complexes (Complex I, II, III, IV, and V) [2]. The ETC proteins are encoded by two distinct genomes: the mitochondrial genome (mtDNA) and the nuclear genome (nDNA). mtDNA are circular, intron-free, and polyploid double-stranded DNA molecules consisting of 16569 nucleotides that encode 13 proteins, 22 transfer RNAs and 2 ribosomal RNAs [3, 4]. Conversely, nDNA encodes for approximately 1500 genes involved in mitochondrial functions and localization [5]. Within ETC, complex I is the largest multimeric protein complex and consists of 45 subunits, from which 7 subunits are encoded by mtDNA [6].

Schizophrenia (SZ) and bipolar disorder (BD) are complex polygenic neuropsychiatric disorders. One pathway implicated in the pathophysiology of SZ and BD is mitochondria dysfunction [5, 7, 8]. Clinical studies have shown that patients with SZ and BD have abnormal energy metabolites and redox states implicating dysfunctional mitochondria [9–11]. Genome-wide association studies (GWASs) and gene-set analysis have linked the pathophysiology of SZ and BD to synaptic dysfunction and nuclear-encoded mitochondria genes (NEMG) [12]. Mitochondrial dysfunction in SZ and BD appears to stem from a complex set of causalities (a) single nucleotide polymorphism (SNP) in mtDNA and NEMGs [13]; (b) decreased expression of genes responsible for mitochondrial function revealed by transcriptomics analysis [14, 15]; (c) altered mtDNA deletion burdens in SZ and BD brain tissues [16, 17]; (d) altered mtDNA copy number [18]; (e) altered enzymatic activity of ETC proteins [19]; (f) an altered number of mitochondria and mitochondria volume density [5]. These studies suggest that genetic risk may play a significant role in causing dysfunctional mitochondria in SZ and BD. Furthermore, induced pluripotent stem cells (iPSCs) derived neurons and cerebral organoids from patients with SZ and BD showed dysfunctional mitochondria, which further strengthens the role of complex genetics in the pathophysiology of SZ and BD [20, 21].

Given the complex nature of SZ and BD, we conducted a systematic investigation of critical mitochondria parameters related to mtDNA, and mitochondrial distribution and function, in postmortem brain samples of subjects with SZ and BD. Specifically, we investigated these mitochondrial parameters in clinically well-defined controls, SZ, and BD subjects: mtDNA copy number, mtDNA common deletions, mtDNA total deletions, complex I activity, total mitochondria number, total synapse number, and the number of synaptic mitochondria. The neuronal circuit between the prefrontal cortex (PFC) and nucleus accumbens (NAc) plays a crucial role in reward and emotional behavior processing [22]. Clinical studies have identified that impaired signaling within PFC and NAc is one of the hallmarks in the pathophysiology of SZ and BD [23, 24]. Moreover, SZ and BD patients often show abnormal auditory and visual perceptions (our ~93% SZ subjects and ~44% BD subjects had hallucinations; Table ST1B). Both SZ and BD patients have been shown to have decreased gray matter thickness of the superior temporal gyrus (STG, brain region processing auditory perception) [25, 26] and primary visual cortex (V1, brain region processing visual perception) [27, 28]. Thus, the following four key brain regions were investigated to identify the regional effect and specificity of any observed mitochondrial difference in SZ and BD: dorsolateral prefrontal cortex (DLPFC), superior temporal gyrus (STG), primary visual cortex (V1) and nucleus accumbens (NAc).

## Materials and methods

### Subjects

Postmortem brain collection and preservation were conducted as previously published procedures [29]. Briefly, postmortem brain tissues were obtained from the UCI-Pritzker brain bank. Written consent was obtained from the next of kin for each subject. The postmortem brain collection and experimental procedures were approved by UCI Institutional Review Board (IRB). Psychological autopsies were conducted based on family informant interviews, subjects’ medication history, medical and psychiatric records, and coroners’ toxicology reports. Based on psychological autopsies, three clinically well-defined experimental groups were studied: Control (CON), Schizophrenia (SZ) and Bipolar Disorder (BD). In total, 45 CON, 30 SZ, and 27 BD subjects were included in this study. Results for some of these parameters were previously published for DLPFC (parameters copy number, complex I activity, common deletion) with the same subjects [18], and are included for comparisons to three additional brain regions. The demographics of all the subjects have been depicted in Table ST1A. The detailed clinical characteristics of the subjects have been presented in Table ST1B.

### mtDNA copy number and common deletion

The mtDNA copy number (mtDNA CN), mtDNA common deletion (mtDNA CD; adjusted breakpoints:8470-13447), and mtDNA amplicon resequencing was performed on extracted genomic DNA (gDNA) of samples described in our previously published procedures [16, 18] and Supplementary Information.

### Pooled effect of total deletion of mtDNA by high-resolution pipeline “Splice-Break”

With our recently developed Splice-Break pipeline [30], we measured total deletions of mitochondria DNA (common deletion + other deletions), which occur at different rates in different brain regions. The Splice-Break method is described in Supplementary Information and [30]. The deletion data for mtDNA is available through dbGaP accession code: phs002395.v1.p1.

### Complex I activity (activity/unit of Complex I protein)

The complex I activity was measured based on our previously published two-step procedure [18] and Supplementary Information. Our complex I activity assay is not rotenone-based, thus avoids rotenone-based assay disadvantages such as low sensitivity and non-specificity [31].

### Immunohistochemistry (IHC) for colocalization of mitochondria and synaptic marker

The gene TOMM40 (translocase of outer mitochondrial membrane 40) codes for the protein TOM40 which is required to import proteins into the mitochondria. The PSD95 protein (postsynaptic density protein 95) is exclusively localized in excitatory synapses. The colocalization of TOM40 and PSD95 was evaluated by immunohistochemistry (IHC) as described previously [32–34] with slight modification described in Supplementary Information.

### Statistical analyses

The raw data for each dependent variable was imported into JMP Pro 16.0 (SAS Institute, Inc., NC, USA) and checked for outliers greater than 3 standard deviations from the mean, which were removed. Additional variables beyond diagnosis and brain region (age, PMI, pH, sex) were checked for correlations with raw data and were included in models when significant. Linear regression models for single mitochondria variables were used to determine group differences in a single brain region. When similar technology was used across three cortical regions in which all subjects were assayed, a repeated measure (mixed effects linear model) based on subject, region, and the diagnosis was calculated to increase the analysis robustness compared with single region analysis. Post hoc group effects were analyzed after main effects or interaction effects were determined significant (*p* < 0.05). For mtDNA CD, mtDNA deletions per 10 K coverage and complex I activity: all three cortical areas (DLPFC, STG, and V1) were analyzed together for regional correction. In contrast, NAc was analyzed separately due to non-cortical area and its low sample size. For mtDNA CN, the DLPFC and STG were analyzed together for regional correction, while V1 and NAc were analyzed individually because the DLPFC and STG mtDNA CN were obtained through qPCR, while V1 mtDNA CN was obtained through ddPCR. Exploratory analysis of medication effects used previous grouping of subjects with neuroactive compounds in cerebellar toxicology reports from NMS Labs (PA, USA) as a grouping variable in pooled BD and SZ subjects. The details of these subjects’ toxicology were previously published [18], and a summary of the toxicology findings is presented in Supplementary Table ST1B.

## Results

### mtDNA copy number in SZ and BD

**Fig. 1:**
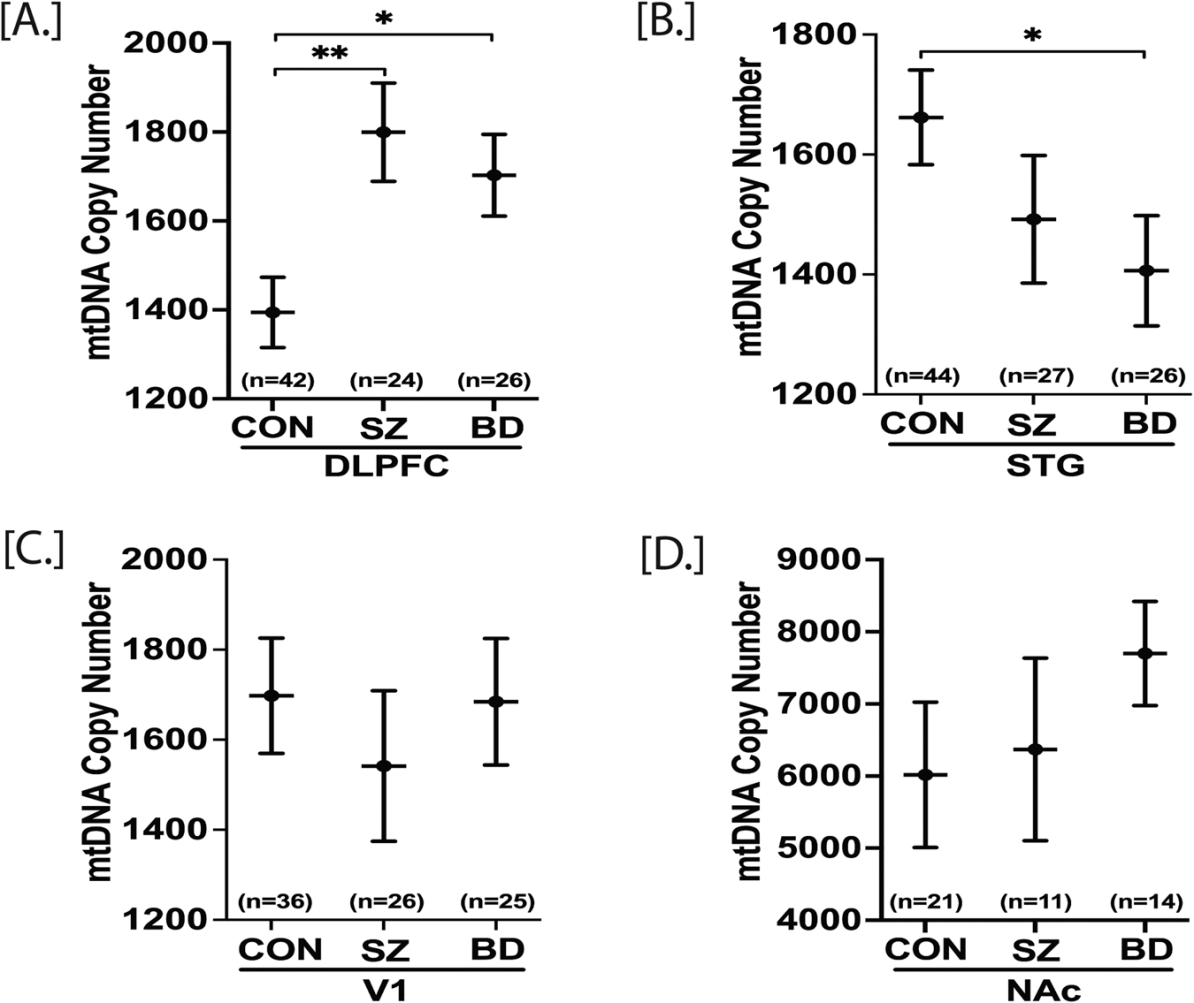
mtDNA copy number in postmortem DLPFC, STG, V1 and NAc of control (CON), schizophrenia (SZ) and bipolar disorder (BD) groups. mtDNA copy number (CN) for DLPFC, STG and NAc were obtained through qPCR while V1 mtDNA copy number (CN) was obtained through ddPCR. mtDNA CN of a sample was calculated as mtDNA CN/ALB CN. A repeated measure analysis was performed for statistical significance with adjustment of age and sex for the DLPFC and STG together, whereas V1 and NAc data were analyzed individually. DLPFC mtDNA CN was significantly higher in both SZ and BD compared to control group (**A**). In STG, BD group had significantly lower mtDNA CN compared to control (**B**). In V1 and NAc, there were no significant groups differences (**C**, **D**). All data are represented as least square mean (LSM) ± Std. error. *p* < 0.05 and *p* < 0.01 are denoted by * and **, respectively, otherwise *p* > 0.05.

Decreased peripheral mtDNA CN has been reported in SZ and BD [35, 36]. Peripheral mtDNA CN has also been shown to decrease with aging, although brain tissue mtDNA CN showed no age-related changes [37, 38]. Here, we sought to determine how SZ and BD affect the mtDNA CN in three cortical brain regions [DLPFC, STG, V1] and in the ventral striatum (NAc). As a repeated analysis, a mixed model effects analysis (~age+sex+region+subject) was conducted for mtDNA CN in the DLPFC and STG regions. The V1 and NAc regions were separately analyzed because V1 data were obtained through ddPCR, and NAc regions had a subset of samples. For the DLPFC, both SZ and BD groups displayed significant increases (*p* = 0.003 and *p* = 0.011, respectively) in the level of mtDNA CN compared to CON (Fig. 1A). The same subjects’ STG region showed a significant decrease (*p* = 0.036) in mtDNA CN in BD compared to controls and a non-significant decrease in mtDNA CN in SZ compared to controls (Fig. 1B–D) (Table ST2). Although the interaction of the brain region (DLPFC, STG) and the diagnosis was significant (*p* = 0.0015), the mtDNA CN brain region correlations (DLPFC vs. STG) were not significant (Fig. SF1).

In separate analyses of V1 cortex and nucleus accumbens regions, there were no significant differences between groups for mtDNA CN in the V1 (*p* = 0.72) or the NAc (*p* = 0.30). Next, we determined the effect of age on mtDNA CN in DLPFC, STG, and V1. Age did not significantly affect mtDNA CN in DLPFC, STG, and V1 (Table ST7). Another potential confounding factor affecting mtDNA CN in SZ and BD is the use of antipsychotic and other medications shown to decrease peripheral mtDNA CN [39]. Further, we addressed if correlations of the mtDNA CN were altered by antipsychotic drugs and other medications. For this, patients were grouped by detectable or not detectable toxicological psychotropic drugs and medications based on postmortem toxicology analysis. The presence or absence of medications was based on toxicological results performed on postmortem brain tissues (Table ST1B). Thus, we cannot exclude the possibility of previous exposures to medications having an effect. We found that the presence of medications at the time of death had no significant effect on mtDNA CN in four brain regions included in this study (Table ST8), suggesting these brain regions may have more stable mitochondrial CN than blood when exposed to environmental factors or medication. MtDNA CN was ~400% higher in NAc compared to the three cortical regions [DLPFC, STG, and V1] in all three groups [CON, SZ, and BD] (Fig. 1, Table ST2).

### Common deletion of mtDNA in SZ and BD

**Fig. 2:**
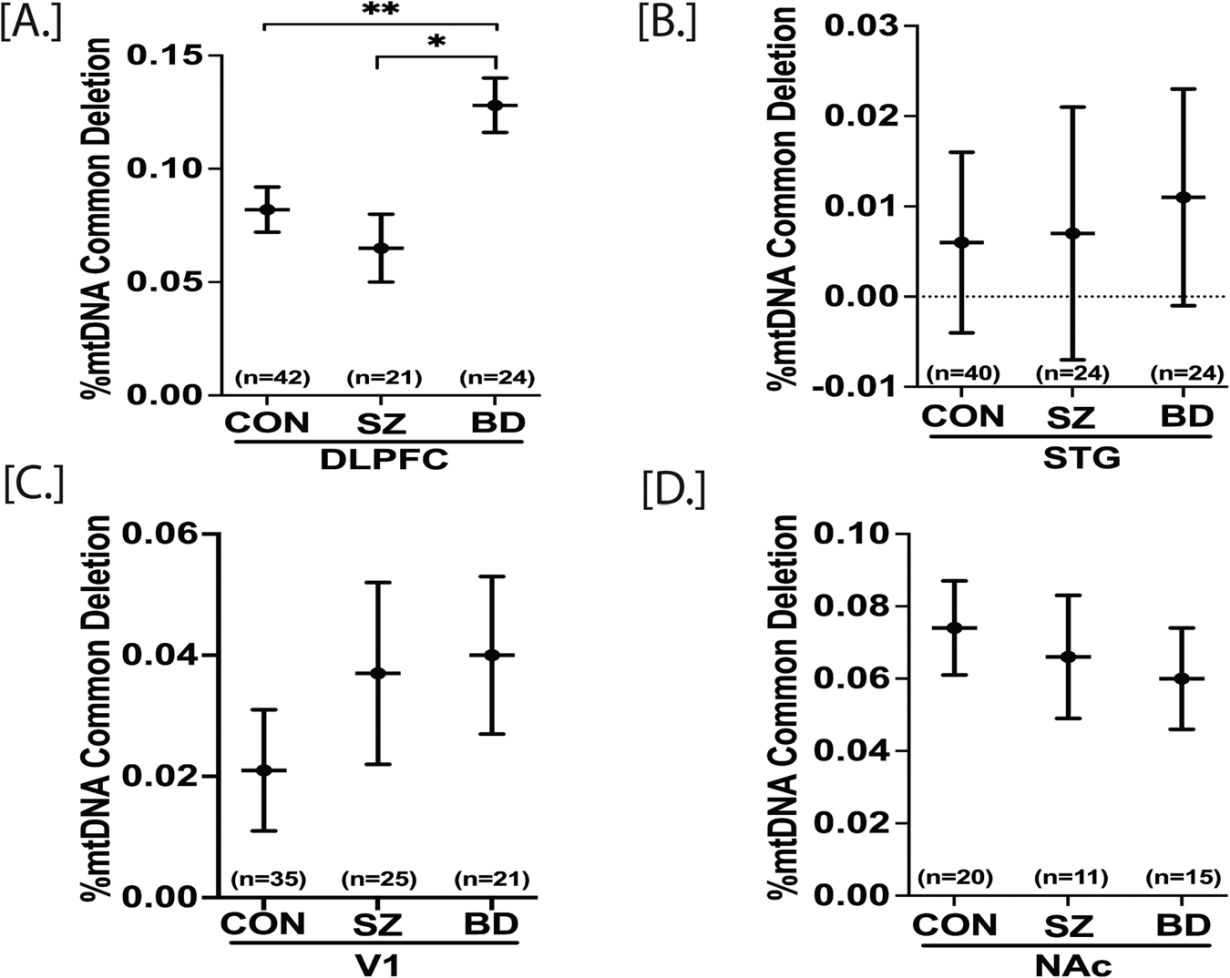
mtDNA common deletions in postmortem DLPFC, STG, V1 and NAc of control (CON), schizophrenia (SZ) and bipolar disorder (BD) groups. mtDNA common deletion (CD) for DLPFC, STG and NAc were obtained through qPCR while V1 mtDNA copy number was obtained through ddPCR. %mtDNA CD was calculated as [mtDNA CD copies/ (wild type mtDNA copies+ mtDNA CD copies)] x 100. A repeated measure analysis was performed for statistical significance with adjustment of age and sex for the DLPFC and STG. The V1 and NAc were analyzed separately. DLPFC mtDNA CD was significantly higher in BD compared to control group (**A**). There were no significant group differences in STG, V1 and NAc mtDNA CD (**B**–**D**). All data are represented as least square mean (LSM) ± Std. error. *p* < 0.05 and *p* < 0.01 are denoted by * and **, respectively, otherwise *p* > 0.05.

The mtDNA common deletion (CD) is a large (4977 bp) deletion of mtDNA, shown by us and others to increase with age in multiple brain regions [16, 40]. The mtDNA CD is an indicator of oxidative stress due to reactive oxygen species (ROS) and the loss of 12 essential mitochondrial genes within the deleted region. mtDNA is situated near the ETC, a major source of ROS that can damage mtDNA and trigger deletions. A mixed model analysis was performed for statistical significance with adjustment of age and sex. The DLPFC, STG, and V1 data were analyzed together, whereas NAc data were analyzed individually. The mtDNA CD was increased in BD compared to CON (*p* = 0.014) and increased in BD compared to SZ (*p* = 0.046) in the DLPFC (Fig. 2A). The %CD in STG, V1, and NAc was not significantly altered (Fig. 2B–D) (Table ST3).

In contrast to the mtDNA CN results, the mtDNA CD showed highly significant correlations in the levels between brain regions. Regional analyses of the CD data showed a significant age effect (%CD increases with age) in each of the three cortical regions (Fig. SF3). The positive correlation of age and the CD was significant in V1 cortex (*p* = 0.032, *r* = 0.22), DLPFC (*p* = 0.0008, *r* = 0.34), and STG (*p* = 0.008, *r* = 0.27) (Table ST9). The positive correlations of the mtDNA CD were highly significant across regions by subject, e.g., a high inter-regional correlation (STG-DLPFC, r = 0.75, 3.25E−18), which suggests a possible global mechanism for the common deletion dependent upon age (Fig. SF2). A subset of subjects was assayed for the mtDNA CD in the NAc and the inter-regional correlations remained significant when analyzing only this subset of subjects across 4 brain regions with common subjects assayed in all four regions. Further, we addressed if correlations of the mtDNA CD were altered by antipsychotic drugs and other medications (based on toxicology reports). The inter-regional correlations remained significant for both groups regardless of toxicology measures. Thus, the presence of medications at the time of death did not alter the significant inter-regional correlations of mtDNA CD levels, suggesting it either had no effect, or had an equivalent and non-significant effect on four brain regions evaluated (data not shown).

### The effect of total mtDNA deletions in SZ and BD

**Fig. 3:**
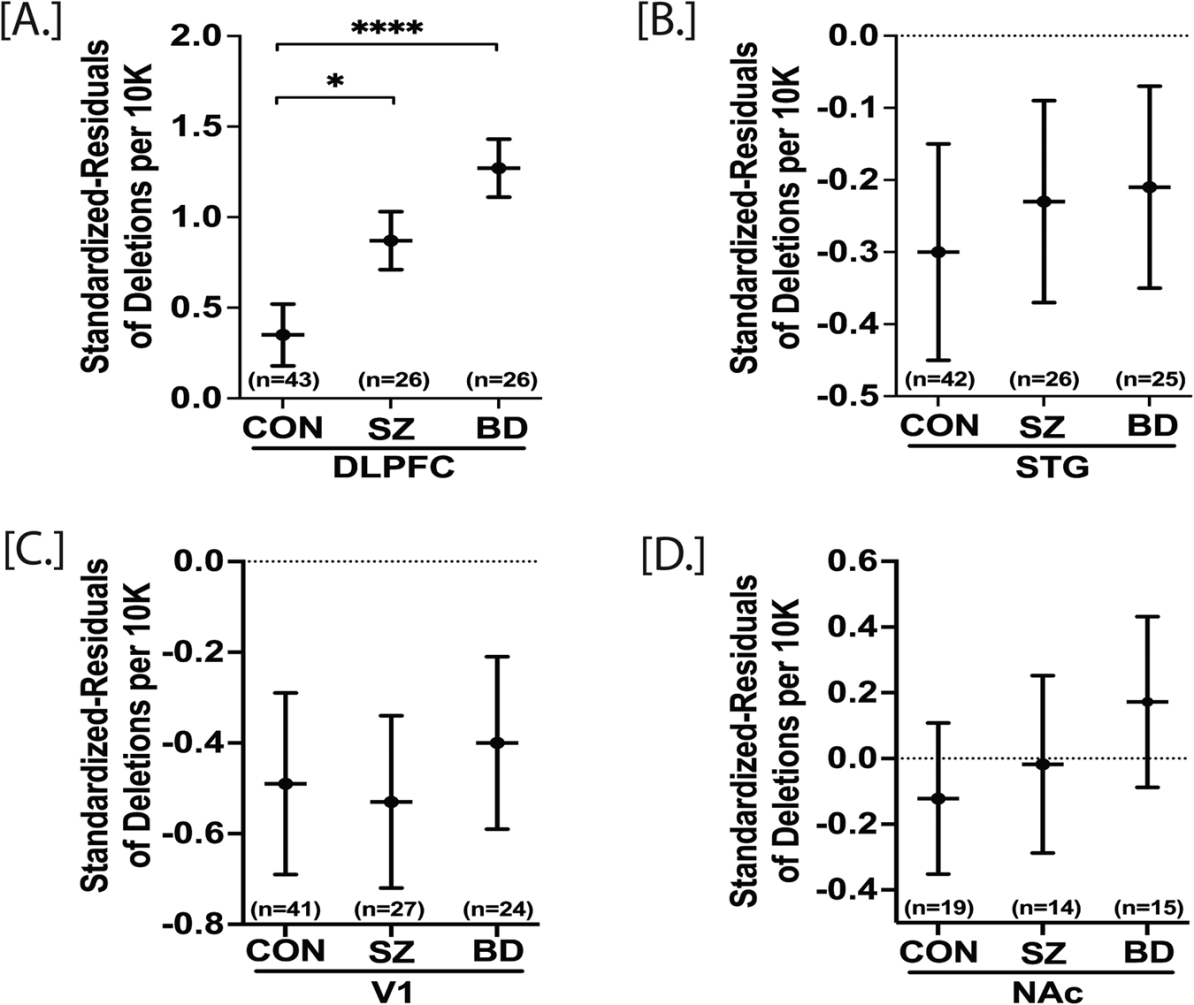
Standardized residuals of deletions per 10 K in postmortem DLPFC, STG, V1, and NAc of control (CON), schizophrenia (SZ), and bipolar disorder (BD) groups. A repeated measure analysis was performed for statistical significance with adjustment of age and sex. DLPFC, STG, and V1 were analyzed together to obtain the regional correction, whereas NAc data were analyzed individually. DLPFC standardized residuals of deletion per 10 K were significantly higher in both SZ and BD compared to control group (**A**). There were no significant group differences in STG, V1, and NAc (**B**–**D**). Deletion levels are negative based on standardized residuals. All data are represented as least square mean (LSM) ± Std. error. *p* < 0.05 and *p* < 0.0001 are denoted by * and ****, respectively, otherwise *p* > 0.05.

We utilized the highly sensitive “Splice-Break” pipeline to detect the total number of unique mtDNA deletions whose breakpoints fell within the position of mtDNA 357-15925. The “Splice-Break” pipeline provided us deletion read %’s for hundreds to thousands of mtDNA deletion breakpoints, and we calculated the cumulative deletion metrics as previously described [30]. Here, we focused on the cumulative analysis of “deletions per 10K coverage”, which is the estimation of the pooled effect of total deletions species (i.e., unique set of breakpoints), as the other two metrics described in our method [30] were not significantly different in group comparisons. Previously, we showed that deletions per 10K coverage parameter did not significantly vary with age, brain pH and postmortem interval (PMI) [30]. Here, we computed standardized residuals for deletions per 10K coverage with benchmark coverage and age added as a covariate. The repeated measure analysis revealed that the standardized residuals of deletions per 10K were significantly higher in DLPFC of both SZ (*p* = 0.036) and BD (*p* = 0.000055) compared to CON (Fig. 3A). There were no other significant group comparisons within STG [CON vs SZ, *p* = 0.78; CON vs BD, *p* = 0.68], V1 [CON vs SZ, *p* = 0.87; CON vs BD, *p* = 0.71] and NAc [CON vs SZ, *p* = 0.77; CON vs BD, *p* = 0.40] (Fig. 3B–D) (Table ST4). The deletions per 10K coverage were not significantly correlated with age (Table ST10). The presence of medications at the time of death did not affect the total deletions per 10K coverage in DLPFC, STG, and V1 (Fig. SF4).

### Complex I activity in SZ and BD

**Fig. 4:**
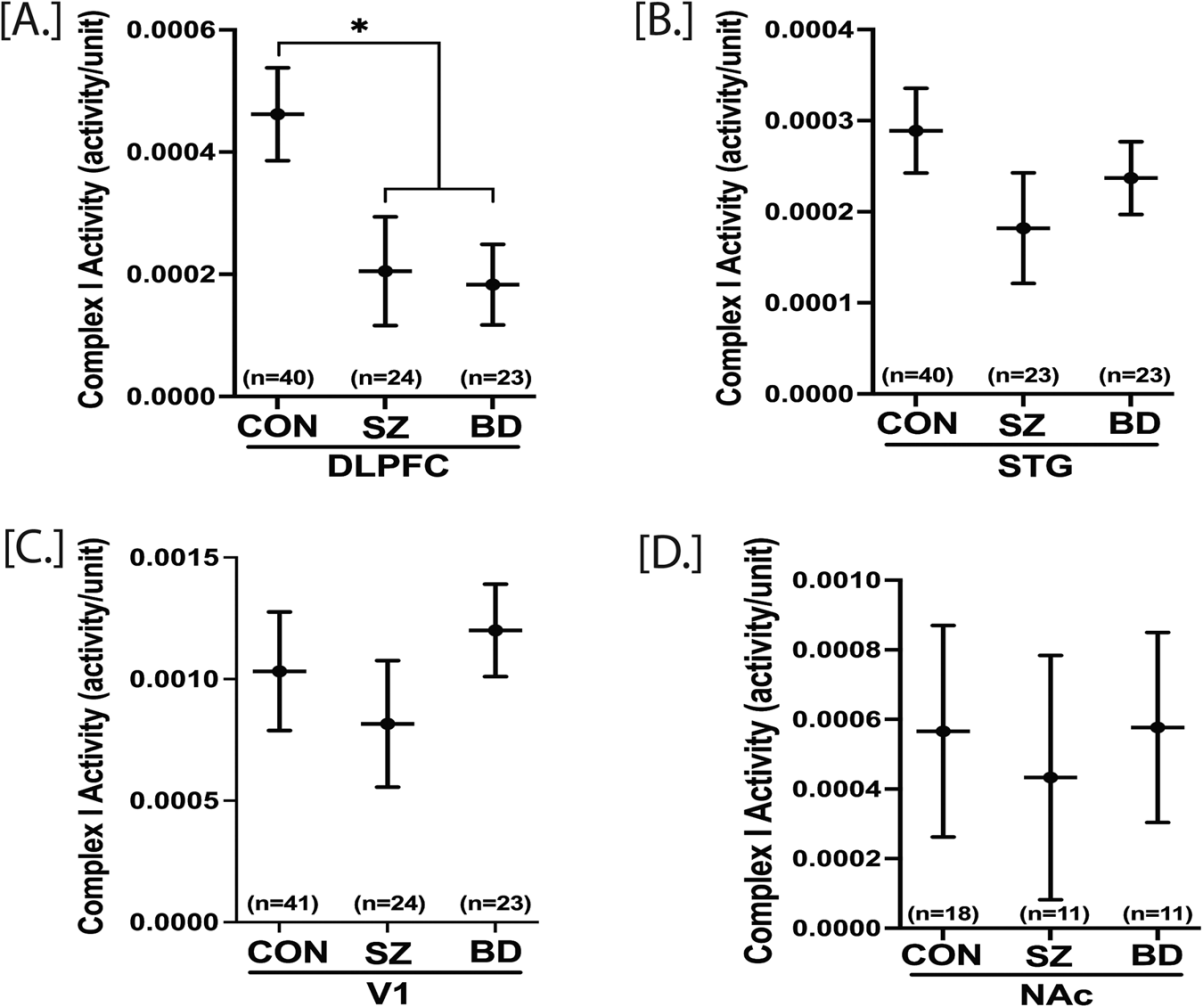
Complex I activity in postmortem DLPFC, STG, V1 and NAc of control (CON), schizophrenia (SZ), and bipolar disorder (BD) groups. A repeated measure analysis was performed for statistical significance with adjustment of age and sex. DLPFC, V1 and STG complex I activity were analyzed together, whereas NAc complex I activity was analyzed individually. DLPFC complex I activity was significantly lower in both SZ and BD compared to the control group (**A**). There were no significant group differences in STG, V1, and NAc (**B**–**D**). All data are represented as least square mean (LSM) ± Std. error. *p* < 0.05 is denoted by *, otherwise *p* > 0.05.

Complex I (NADH ubiquinone oxidoreductase) is the first enzyme complex for cellular oxidative phosphorylation and a major contributor to the proton gradient across the ETC. It has been shown that mitochondria quality decreases with aging, which in turn decreases the complex I activity in various tissues, including the frontal cortex and muscle [18, 41]. Here, we determine if schizophrenia and bipolar disorder complex I activity in four brain regions is altered with adjustment of age and sex. Repeated measure analysis was performed with DLPFC, STG, and V1 regions together, while NAc data were analyzed separately. The complex I activity is significantly decreased in DLPFC of both SZ (*p* = 0.028) and BD (*p* = 0.012) groups compared to CON. SZ and BD did not affect the complex I activity in STG, V1, and NAc (Fig. 4) (Table ST5). The complex I protein concentration, measured by ELISA, was also significantly decreased across the three cortical brain regions (DLPFC, STG, and V1) in both SZ and BD (Fig. SF6) compared to CON group (*p* < 0.01). The complex I protein concentration in NAc was not significantly different among groups.

The regional correlation of complex I activity was not significant across DLPFC, STG, V1, and NAc (Table ST11). The STG complex I activity was dependent on age, while DLPFC, V1, and NAc complex I activity was independent of age (Table ST11). An analysis of medications/drug (present at the time of death) effects on complex I showed that the DLPFC complex I activity was decreased (*p* = 0.0073) (Table ST12) by medications. At the same time, there was no medications/drug effect on STG, V1, and NAc complex I activity (Table ST12). Thus, the effects of medications/drugs may be regionally specific. We further explored the medication effect on DLPFC complex I activity. We pooled SZ + medication subjects with BD + medication subjects (SZ/BD + Med), and SZ-medication subjects with BD-medication subjects (SZ/BD-Med). DLPFC complex I activity was significantly lower in SZ/BD + Med group compared to the CON group (Fig. SF7). However, DLPFC complex I activity was decreased, but not significantly different between SZ/BD-Med and CON groups (*p* = 0.16, Fig. SF7). We also determined the correlation of standardized residuals of deletions per 10 K coverage with complex I activity. The correlation was not statistically significant (*p* = 0.3081) across the three cortical brain regions (DLPFC, STG, and V1) (Fig. SF5).

### Overall mitochondria number, synapse number, and synaptic mitochondria number in SZ and BD

**Fig. 5:**
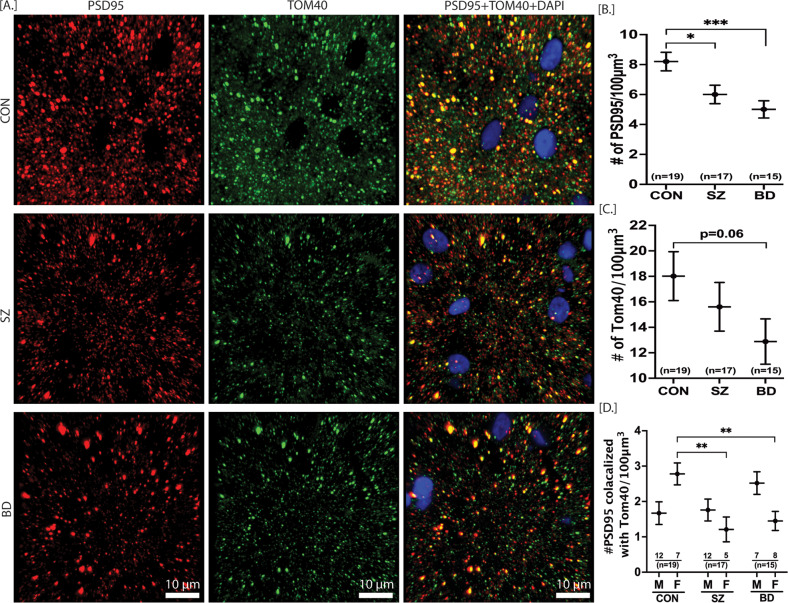
Overall mitochondria number, synapse number and synaptic mitochondria number in DLPFC of CON, SZ and BD subjects. The representative images for PSD95, TOM40 and PSD95 colocalized with TOM40 are depicted in (**A**). A repeated measure analysis was performed for statistical significance with adjustment of age and sex. The number of PSD95 was significantly lower in both SZ and BD compared to CON group (**B**). The overall number of TOM40 was not significantly different in SZ and BD compared to CON, although there was a decrease trend in BD (**C**). The sex x group effect was significant for the PSD95 colocalized with TOM40. Both the female SZ and female BD subjects had significantly lower PSD95 colocalized with TOM40 compared to CON females [**D**]. All data are represented as least square mean (LSM) ± Std. error. *p* < 0.05, *p* < 0.01 and *p* < 0.001 are denoted by *, ** and ***, respectively, otherwise *p* > 0.05. CON = control (*n* = 19), SZ = schizophrenia (*n* = 17), BD = bipolar disorder (*n* = 15), Red spots = PSD95, green spots = TOM40, yellow spots = PSD95 colocalized with TOM40, blue = DAPI nuclear stain.

The synaptic strength and synaptic activity depend on ATP production by mitochondria [42]. Thus, mitochondria play a pivotal role in synaptic neurotransmission. Among four brain regions studied here, the DLPFC has been the most affected area in terms of mtDNA CN, mtDNA deletions, and complex I activity. Therefore, we investigated any change in overall mitochondria number, synapse number, and synaptic mitochondria number in DLPFC of SZ and BD subjects. The overall number of mitochondria, synapse, and synaptic mitochondria was determined through immunostaining of mitochondria surface marker TOM40, synaptic marker PSD95, and colocalization of TOM40 + PSD95, respectively. The mixed model analysis was conducted with adjustment of subjects’ age, pH, and sex. For the overall synapse number (# of PSD95 puncta), the main effect of the diagnosis group was significant. The synaptic number marker (number of PSD95) was significantly lower in the DLPFC of both SZ and BD (*p* = 0.0008) subjects compared to control subjects (Fig. 5B). There was no significant main effect of group for overall mitochondria number (# of TOM40). TOM40 was not significantly different in SZ (*p* = 0.388) and BD (*p* = 0.065) groups compared to the CON group. For the synaptic mitochondria number (colocalized TOM40 with PSD95), the main effect of the group was not significant. However, very interestingly, the sex x group interaction was statistically significant. The female CON group had significantly higher (*p* = 0.013) synaptic mitochondria compared to the male CON group (Table ST6). Both the female SZ (*p* = 0.002) and female BD (*p* = 0.003) groups had significantly lower synaptic mitochondria compared to the female CON group (Table ST6). The male subjects in the SZ and BD groups did not differ from male CON subjects for synaptic colocalized mitochondria (*p* = 0.85, *p* = 0.09, respectively) (Fig. 5C–E) (Table ST6). The age did not significantly affect the number of PSD95, TOM40, and colocalized PSD95 with TOM40 (Table ST13). Similarly, the number of PSD95, TOM40, and colocalized PSD95 with TOM40 was not dependent on medications present at the time of death (Table ST14).

## Discussion

### Regional alteration of mtDNA copy number in SZ and BD

Subjects with schizophrenia showed increased mtDNA CN in DLPFC with no alterations of mtDNA CN in STG, V1, and NAc regions. On the other hand, bipolar patients showed increased mtDNA CN in DLPFC, decreased mtDNA CN in STG, and unaltered mtDNA CN in V1 and NAc (Fig. 1). These mtDNA CN results suggest that although schizophrenia and bipolar disorder have overlapping effects on mtDNA CN in various brain regions, it is not uniform for all brain regions. The decreased mtDNA CN has also been reported in the postmortem hippocampus of BD patients [43]. Interestingly, peripheral mtDNA CN showed a marked decrease in both SZ [36, 44, 45] and BD [35, 46–48], in contrast to our postmortem DLPFC mtDNA CN marked increase. This discrepancy might be because the brain tissue has a higher bioenergetics profile than peripheral tissue. There were significant differences in mtDNA CN between regions, with NAc having 400% higher mtDNA CN than cortical regions. This higher bioenergetic demand in the NAc suggests potential regionally targeted approaches relevant to the brain function of this region. Aging is one of the confounding factors affecting peripheral mtDNA CN. Peripheral mtDNA CN has been shown to decrease with aging [49]. Here, we have not found any effect of age on mtDNA CN in all four brain regions, although a trend for decrease with age was observed and warrants further investigation in larger cohorts (Table ST7). These results suggest that aging affects the mtDNA CN parameter in a tissue-specific manner. In both SZ and BD, the mtDNA copy number changes across brain regions are independent, agreeing with other lab reports [50]. The lack of correlation of mtDNA copy number in various brain regions suggests varying energetic demands in each region and perhaps variation in cell types. The mtDNA CN changes in this study were also independent of psychotropic medications or drugs in the postmortem brain (Table ST8).

### The mtDNA common deletion is increased in DLPFC of BD

The mtDNA CD occurs due to various events, including mtDNA replication/repair, oxidative stress, exposure to ionizing radiation, and mitochondria disease [51, 52]. The mtDNA CD can also accumulate as a somatic heteroplasmic deletion with age (positively correlates with age) [51, 52]. The age-related accumulation rate of mtDNA CD is tissue-specific and more pronounced in tissues with higher energy demands [30, 51, 52]. Here we sought to determine schizophrenia- or bipolar disorder-specific alteration of mtDNA CD, and at the same time, if there was any region-specific effect. We find that the mtDNA CD of SZ subjects remains unaltered in all four brain regions (DLPFC, STG, V1, and NAc). On the other hand, mtDNA CD is increased in DLPFC of BD subjects and is unaltered in STG, V1, and NAc (Fig. 2). The increased mtDNA CD in DLPFC of BD subjects is in accordance with previous findings from our lab determined with independent samples [17]. Previously, we showed a global decrease of mtDNA CD in SZ subjects across 10 brain regions [16], including cortical and striatal regions. In contrast to the global effect previously reported across10 brain regions in SZ, the mtDNA CD was analyzed here in four brain regions, thus reducing the statistical power. The unaltered cortical mtDNA CD in SZ mirrors the previously published results from our and other labs [17, 40, 53].

### The total mtDNA deletions are increased in DLPFC of both SZ and BD subjects

Previously we showed that the mtDNA common deletion was neither the most frequent nor the most abundant deletion in human brain tissues [30]. This observation prompted us to explore the burden of total somatic deletions of mtDNA in schizophrenia and bipolar disorder. Here, we report that the total deletion burden (residuals of deletion per 10 K coverage) is increased in the DLPFC of both SZ and BD subjects (Fig. 3). The residuals of deletion per 10 K were not significantly altered in STG, V1, and NAc of both SZ and BD subjects. This finding is also consistent with our preliminary observation that the cumulative deletion burden of the DLPFC, relative to the anterior cingulate cortex, was increased in a group of SZ and BD subjects when compared to subjects with no psychiatric disease, depression or alcohol dependency [30]. As expected, the deletions per 10 K coverage were independent of age and medications in all four brain regions (Table ST10, Fig. SF4). Overall, the effects of illness induced stressors such as profound sleep disturbance, altered diet, altered metabolism and microbiome, and others could play a substantive role in mitochondria deletions. The majority of SZ and BD subjects had anxiety and depressed mood. Thus, the increased mtDNA deletions in the DLPFC of SZ and BD subjects could also be due to anxiety and depression rather than direct pathological etiology. In contrast, the observed mtDNA deletions could be upstream effectors of SZ and BD symptoms. Moreover, it remains equally likely that mitochondrial defects could be downstream of incipient etiology and initial symptoms but still influence illness course and quality of life.

### Decreased Complex I activity in DLPFC of both SZ and BD

We have used a dual method to measure complex I activity that takes into account the amount of complex I protein present, then equalizes that amount in the second step of the assay and measures the conversion of NAD + to NADH. This measure is analogous to a specific activity per unit of enzyme. We also tested for protein abundance deficits prior to normalizing values of complex I protein for activity measures. The mtDNA CD removes 7 genes encoding 4 complex I subunits. Thus, we expected decreased complex I activity in the DLPFC of BD subjects since mtDNA CD and mtDNA deletions per 10k was increased in DLPFC of BD subjects (Fig. 2). As expected, the complex I activity was significantly decreased in DLPFC (with no alteration in STG, V1, and NAc of BD subjects, Fig. 4). Although the mtDNA CD was unaltered in DLPFC of SZ subjects (Fig. 2), we found mtDNA deletions per 10k was increased in DLPFC in SZ, and complex I activity was also decreased in DLPFC (with no alteration in STG, V1, and NAc of SZ subjects (Fig. 4). Thus, the increased rate of total mtDNA deletion in DLPFC of SZ subjects, could account for decreased complex I, however, there were no significant correlations between complex I activity and the residual deletions per 10k in any brain region (Fig. SF5). The decreased DLPFC complex I activity markedly contrasts with the peripheral platelet complex I activity of SZ (active psychosis) and BD subjects, where the complex I activity is significantly increased [54–56]. Thus, different molecular mechanisms in blood and the brain might affect the peripheral and brain complex I activity. The recent meta-analysis [41] showed that the SZ and BD groups had moderate (statistically insignificant) effects on complex I activity. This discrepancy might be attributed to the regional heterogeneity of alteration in complex I activity by SZ and BD. Our postmortem complex I findings align with previously published reports showing decreased complex I activity in DLPFC and temporal cortex of SZ and BD subjects [18, 19, 57, 58].

The amount of complex I protein was decreased across the three cortical regions (DLPFC, STG, and V1) in both schizophrenia and bipolar disorder compared to the CON group. This finding supports other work showing decreased complex I subunits in the brain of SZ and BD subjects [41]. The presence of medications at the time of death had region-specific effects on complex I activity. The presence of medications decreased DLPFC complex I activity without affecting the complex I activity in STG, V1, and NAc (Table ST12). The decreased DLPFC complex I activity in the presence of medications agrees with previous in-vitro and rodent model reports showing decreased complex I activity induced by antipsychotics [59, 60]. The DLPFC complex I activity in SZ/BD-Med group (medication-free at the time of death) was reduced by 25% but not significantly altered compared to the CON group (Fig. SF7). This result signifies that the decreased DLPFC complex I activity in SZ/BD groups is driven by the presence of various medications due to the mixing of medications and drugs of abuse. However, it is not possible to ascertain which compounds affected complex I. These findings need to be verified with larger sample size, with a more restricted medication toxicology screen, as our sample size in pooled SZ/BD-Med group (medication-free at the time of death) was 12. Another potential issue is that patients requiring medication might have had more severe illnesses than medication-free patients. Although medications can play a role, there are multiple ways complex I activity might be altered in the DLPFC. Large deletions of mtDNA are increased in the DLPFC of both SZ and BD, creating functional mutations in complex I and other complexes. It is being studied whether these large deletions are transcribed and translated. A decreased complex I activity could also be due to assembly defects in both mtDNA and nDNA encoded complex I subunits, reducing both protein amount and activity.

### Loss of synapses and sex-specific loss of synaptic mitochondria in both SZ and BD subjects

The energy from mitochondria is utilized in neuronal communication such as the development of cytoskeleton for presynapse development, synaptic vesicle transport, and generation of synaptic membrane potentials [61, 62]. Mitochondria also play a crucial role in synaptic neurotransmission through intracellular Ca^2+^ buffering [62]. Thus, the number of mitochondria remains higher in high-energy demanding sites such as axon terminals and postsynaptic areas compared to other parts of the neurons [62]. Fewer mitochondria number and/or dysfunctional mitochondria would lead to fewer synapse number or aberrant synaptic communication. By immunohistochemistry, we identified any alteration of the overall mitochondria number, synapse number, and synaptic mitochondria number in SZ and BD after adjustment of age and sex. We find that the overall synapse number are significantly decreased in DLPFC of both SZ and BD subjects. This is an important cellular feature in the pathophysiology of SZ and BD, that mimics recent studies showing the decrease of excitatory synapses or postsynaptic elements in the prefrontal cortex and anterior cingulate cortex of schizophrenia subjects [63–65].

In contrast to the synapse number, the overall mitochondria number (as measured by IHC that was not restricted to excitatory synapses) was not significantly altered in SZ and BD compared to controls. Decreased mitochondria number has been reported in the axon terminals and layer 3 excitatory synapses of anterior cingulate cortex [58, 64, 66], and in the oligodendrocytes of prefrontal cortex of SZ subjects [67, 68]. This discrepancy might be attributed to the fact that our calculated overall mitochondria belong to all types of cells, multiple layers of cortex, and not restricted to high energy demanding sites such as axon terminals or synapses. Our finding of unaltered mitochondria number in DLPFC is in concert with another report showing no change of mitochondria number or size in NAc of subjects with SZ [23]. To calculate the synaptic mitochondria number, we measured the colocalized spots for mitochondria marker TOM40 with synapse marker PSD95. Interestingly, we have found that synaptic mitochondria number are decreased in both female SZ and female BD groups compared to female control group. This finding warrants future investigation with a larger sample size as a low sample size is an obvious limitation of this finding to be further tested in other brain regions.

## Conclusion

Among the four brain regions, the DLPFC remains the most affected brain region in terms of mitochondrial parameters studied. The mitochondria unique deletions and copy number are both increased, while complex I activity is decreased in the DLPFC of both schizophrenia and bipolar subjects compared to controls. The decreased DLPFC complex I activity was driven by the presence of medications at the time of death as unmedicated SZ and BD subjects (pooled) did not show significantly reduced complex I activity. The amount of complex I protein was decreased across three cortical regions in both schizophrenia and bipolar disorder. Decreased excitatory synapses in schizophrenia and bipolar disorder are relevant to these findings. These indicators of mitochondria dysfunction require further investigations into cell specificity of the findings and potential relationship to genetic influences to understand the pathophysiology of schizophrenia and bipolar disorder.

## Supplementary information

Supplementary Information

Supplementary Table ST1B
