# Supplementary material for: Mitochondria DNA copy number, mitochondria DNA total somatic deletions, Complex I activity, synapse number, and synaptic mitochondria number are altered in schizophrenia and bipolar disorder
Source: Transl Psychiatry. 2022 Aug 30;12:353. doi: 10.1038/s41398-022-02127-1 (PMC9427957; doi:10.1038/s41398-022-02127-1)
Supplement: Supplementary file 1 — Supplementary Information [file 41398_2022_2127_MOESM1_ESM.pdf]

**Supplementary Information**

Mitochondria DNA copy number, mitochondria DNA total somatic deletions, Complex I activity, synapse number, and synaptic mitochondria number are altered in schizophrenia and bipolar disorder

Das, Sujan C.[1], Hjelm B.E.[2], Galke B.[1], Sequeira A.[1], Morgan L.[1], Omidsalar A.A.[2], Schatzberg, A.F.[3], Barchas, J.D. [4], Lee, F.S.[4], Myers, R.M.[5], Watson, S.J.[6], Akil, H.[6], Bunney, W.E. [7], Vawter M.P.[1#]

**Table ST1A:** Demographics of subjects

| **A. Demographics of three groups analyzed for complex I activity, mtDNA common deletion level, mtDNA copy number, and mtDNA total deletion in DLPFC, STG, and V1 cortex.** | | | | |
|---|---|---|---|---|
| | | | | |
| **Sex** | | **BD** | **CON** | **SZ** |
| Female (N) | | 13 | 6 | 3 |
| Male (N) | | 13 | 35 | 24 |
| | F : M | 1:1 | 1:5.8 | 1:8 # |
| pH | Mean | 6.68* | 6.86 | 6.64* |
| | Std Dev | 0.15 | 0.22 | 0.2 |
| Age | Mean | 46.0* | 57.9 | 41.2* |
| | Std Dev | 12.5 | 12.7 | 10.1 |
| PMI | Mean | 20.5 | 18.8 | 21 |
| | Std Dev | 6.61 | 7.12 | 7.39 |
| # of Meds | Mean | 3.7* | 0.9 | 2.1* |
| | Std Dev | 3.2 | 1.7 | 1.7 |
| | Min | 0 | 0 | 0 |
| | Max | 10 | 10 | 5 |
| # Number of male and female subjects is different between groups | | | | |
| * Significantly different from control group (p < 0.05). | | | | |

| **B. Subset of samples depicted in (A) analyzed for complex I activity, mtDNA common deletion level, mtDNA copy number, and mtDNA total deletion in NAc.** | | | | |
|---|---|---|---|---|
| **Gender** | | **BD** | **CON** | **SZ** |
| Female (N) | | 7 | 3 | 1 |
| Male (N) | | 9 | 18 | 10 |
| All | | 16 | 21 | 11 |
| | **F:M** | **1:1.3** | **1:6** | **1:10** |
| Age | Mean | 43.81*** | 60.76 | 43.00*** |
| Age | Std Dev | 10.51 | 13.91 | 11.8 |
| pH | Mean | 6.71* | 6.87 | 6.63* |
| pH | Std Dev | 0.14 | 0.28 | 0.28 |
| PMI | Mean | 20.82 | 17.72 | 20.96 |
| PMI | Std Dev | 6.84 | 8.53 | 6.94 |
| *** p < 0.0005, ** p < 0.005, * p < 0.05 | | | | |

| **C. Demographics of three groups analyzed for colocalization of TOMM40 and PSD95 in DLPFC.** | | | | |
|---|---|---|---|---|
| **Sex** | | **BD** | **CON** | **SZ** |
| Female (N) | | 8 | 7 | 5 |
| Male (N) | | 7 | 12 | 12 |
| | F : M# | 1:0.88 | 1:1.7 | 1:2.4 |
| pH | Mean | 6.62** | 6.85 | 6.67* |
| | Std Dev | 0.12 | 0.23 | 0.21 |
| Age | Mean | 43.92*** | 64.1 | 43.5*** |
| | Std Dev | 11.92 | 14.32 | 11.97 |
| PMI | Mean | 19.69 | 18.98 | 22.59 |
| | Std Dev | 6.15 | 6.51 | 6.11 |
| # Chi-square statistic 2.99 (N = 51, *p*-value = 0.22) for sex distribution by group.<br>* p < 0.05, ** p < 0.005, *** p < 0.0005, compared to control. | | | | |

**Table ST2:** mtDNA copy number in four brain regions among control, schizophrenia, and bipolar disorder. Repeated measure analysis was conducted based on subject, region, and diagnosis. The DLPFC and STG data were analyzed together. The V1 and NAc data were analyzed individually.

| DLPFC mtDNA Copy Number--qPCR | | | |
|---|---|---|---|
| | Least Square Mean | Std Error | p-Value |
| CON | 1394.3 | 79 | |
| SZ | 1799.5 | 110.4 | 0.003 |
| BD | 1702.9 | 91.9 | 0.011 |

| V1 mtDNA Copy Number--ddPCR | | | |
|---|---|---|---|
| | Least Square Mean | Std Error | p-Value |
| CON | 1697.8 | 127.9 | |
| SZ | 1541.7 | 167.1 | 0.46 |
| BD | 1684.4 | 140.5 | 0.94 |

| STG mtDNA Copy Number--qPCR | | | |
|---|---|---|---|
| | Least Square Mean | Std Error | p-Value |
| CON | 1661.9 | 79 | |
| SZ | 1492.1 | 106.4 | 0.202 |
| BD | 1406.2 | 91.9 | 0.036 |

| NAc mtDNA Copy Number--qPCR | | | |
|---|---|---|---|
| | Least Square Mean | Std Error | p-Value |
| CON | 6016.3 | 1008.2 | |
| SZ | 6369.1 | 1265.5 | 0.82 |
| BD | 7700.4 | 722.1 | 0.17 |

**Table ST3:** mtDNA common deletion in four brain regions among control, schizophrenia, and bipolar disorder. DLPFC, V1, and STG data were analyzed together, whereas NAc was analyzed separately.

| DLPFC mtDNA Common Deletion--qPCR | | | |
|---|---|---|---|
| | Least Square Mean | Std Error | p-Value |
| CON | 0.082 | 0.01 | |
| SZ | 0.065 | 0.015 | 0.36 |
| BD | 0.128 | 0.012 | 0.004 |

| V1 mtDNA Common Deletion--qPCR | | | |
|---|---|---|---|
| | Least Square Mean | Std Error | p-Value |
| CON | 0.021 | 0.01 | |
| SZ | 0.037 | 0.015 | 0.39 |
| BD | 0.04 | 0.013 | 0.24 |

| STG mtDNA Common Deletion--qPCR | | | |
|---|---|---|---|
| | Least Square Mean | Std Error | p-Value |
| CON | 0.006 | 0.01 | |
| SZ | 0.007 | 0.014 | 0.96 |
| BD | 0.011 | 0.012 | 0.72 |

| NAc mtDNA Common Deletion--qPCR | | | |
|---|---|---|---|
| | Least Square Mean | Std Error | p-Value |
| CON | 0.074 | 0.013 | |
| SZ | 0.066 | 0.017 | 0.73 |
| BD | 0.06 | 0.014 | 0.54 |

**Table ST4:** mtDNA deletions per 10K in four brain regions among control, schizophrenia, and bipolar disorder. The DLPFC, V1, and STG data were analyzed together, whereas the NAc was analyzed separately.

| Deletions per 10K-DLPFC | | | |
|---|---|---|---|
| | Least Square Mean | Std Error | p-Value |
| CON | 0.35 | 0.17 | |
| SZ | 0.87 | 0.16 | 0.036 |
| BD | 1.27 | 0.16 | 0.000055 |

| Deletions per 10K-V1 | | | |
|---|---|---|---|
| | Least Square Mean | Std Error | p-Value |
| CON | -0.49 | 0.20 | |
| SZ | -0.53 | 0.19 | 0.867 |
| BD | -0.40 | 0.19 | 0.713 |

| Deletions per 10K-STG | | | |
|---|---|---|---|
| | Least Square Mean | Std Error | p-Value |
| CON | -0.30 | 0.15 | |
| SZ | -0.23 | 0.14 | 0.781 |
| BD | -0.21 | 0.14 | 0.683 |

| Deletions per 10K-Nac | | | |
|---|---|---|---|
| | Least Square Mean | Std Error | p-Value |
| CON | -0.122 | 0.23 | |
| SZ | -0.018 | 0.27 | 0.77 |
| BD | 0.172 | 0.26 | 0.40 |

**Table ST5:** Complex I activity in four brain regions among control, schizophrenia, and bipolar disorder. The DLPFC, V1, and STG data were analyzed together, whereas the NAc was analyzed separately.

| Complex I activity-DLPFC | | | |
|---|---|---|---|
| | Least Square Mean | Std Error | p-Value |
| CON | 0.00046 | 0.00007 | |
| SZ | 0.00020 | 0.00008 | 0.03 |
| BD | 0.00018 | 0.00006 | 0.01 |

| Complex I activity-V1 | | | |
|---|---|---|---|
| | Least Square Mean | Std Error | p-Value |
| CON | 0.0010 | 0.0002 | |
| SZ | 0.0008 | 0.0003 | 0.53 |
| BD | 0.0012 | 0.0002 | 0.61 |

| Complex I activity-STG | | | |
|---|---|---|---|
| | Least Square Mean | Std Error | p-Value |
| CON | 0.00029 | 0.00005 | |
| SZ | 0.00018 | 0.00006 | 0.16 |
| BD | 0.00023 | 0.00004 | 0.43 |

| Complex I activity-Nac | | | |
|---|---|---|---|
| | Least Square Mean | Std Error | p-Value |
| CON | 0.0006 | 0.0003 | |
| SZ | 0.0004 | 0.0004 | 0.77 |
| BD | 0.0006 | 0.0003 | 0.98 |

**Table ST6:** The overall mitochondria number, synapse number, and synaptic mitochondria number in DLPFC of CON, SZ, and BD subjects determined by IHC.

**Group Effect: PSD95**

| Group | LSM | Std Error | p-Value |
|---|---|---|---|
| CON | 8.2 | 0.62 | |
| SZ | 6 | 0.62 | 0.02 |
| BD | 5.01 | 0.57 | 0.0008 |

**Group Effect: Tom40**

| Group | LSM | Std Error | p-Value |
|---|---|---|---|
| CON | 18.02 | 1.92 | |
| SZ | 15.61 | 1.91 | 0.39 |
| BD | 12.88 | 1.78 | 0.06 |

**Group Effect: Coloc**

| Group | LSM | Std Error | p-Value |
|---|---|---|---|
| CON | 2.22 | 0.24 | |
| SZ | 1.49 | 0.24 | 0.04 |
| BD | 1.98 | 0.22 | 0.48 |

**Sex x Group Effect: PSD95**

| Sex | Group | LSM | Std Error | p-Value |
|---|---|---|---|---|
| M | CON | 1.67 | 0.32 | |
| F | CON | 2.78 | 0.31 | |
| M | SZ | 1.76 | 0.31 | 0.54 |
| F | SZ | 1.21 | 0.35 | 0.006 |
| M | BD | 2.52 | 0.32 | 0.07 |
| F | BD | 1.45 | 0.27 | 0.0008 |

**Sex x Group Effect: Tom40**

| Sex | Group | LSM | Std Error | p-Value |
|---|---|---|---|---|
| M | CON | 7.89 | 0.84 | |
| F | CON | 8.52 | 0.81 | |
| M | SZ | 7.1 | 0.81 | 0.96 |
| F | SZ | 4.9 | 0.92 | 0.28 |
| M | BD | 5.49 | 0.84 | 0.21 |
| F | BD | 4.53 | 0.7 | 0.004 |

**Sex x Group Effect: Coloc**

| Sex | Group | LSM | Std Error | p-Value |
|---|---|---|---|---|
| M | CON | 1.67 | 0.32 | |
| F | CON | 2.78 | 0.31 | 0.013 |
| M | SZ | 1.76 | 0.31 | 0.85 |
| F | SZ | 1.21 | 0.35 | 0.002 |
| M | BD | 2.52 | 0.32 | 0.1 |
| F | BD | 1.45 | 0.27 | 0.003 |

**Table ST7:** Correlation of age on mtDNA copy number in DLPFC, STG, V1, and NAc.

| | **DLPFC** | **STG** | **V1** |
|---|---|---|---|
| Pearson r | -0.18009 | -0.20203 | -0.00118 |
| p-Value | 0.087761 | 0.050887 | 0.992 |
| df | 93 | 94 | 86 |

**Table ST8:** Effect of Medications on mtDNA copy number in DLPFC, STG, V1, and NAc.

Group 1 = Pooled BD and SZ subjects positive for neuroactive medications by toxicology screen
Group 2= Pooled BD and SZ subjects negative for neuroactive medications by toxicology screen
Group C = Controls

| | **Group** | | | |
|---|---|---|---|---|
| **Region** | **1** | **2** | **Controls** | **t-test (p-value)** |
| **DLPFC** | | | | **1,2** |
| | | | | |
| Mean | 1691.461 | 1992.11 | 1413.26 | 0.05868 |
| Stdev | 457.7294 | 624.761 | 489.919 | |
| Fold Change | 1.196852 | 1.40958 | | 1, C |
| Group | 1/C | 2/C | | 0.012616 |
| | | | | 2, C |
| | | | | 0.000461 |

| | **Group** | | | |
|---|---|---|---|---|
| **Region** | **1** | **2** | **Controls** | **t-test (p-value)** |
| **STG** | | | | **1,2** |
| | | | | |
| Mean | 1495.799 | 1430.379 | 1538.43 | 0.521886 |
| Stdev | 326.4067 | 356.95 | 421.657 | |
| Fold Change | 0.97229 | 0.929767 | | 1, C |
| Group | 1/C | 2/C | | 0.621633 |
| | | | | 2, C |
| | | | | 0.363372 |

| | **Group** | | | |
|---|---|---|---|---|
| **Region** | **1** | **2** | **Controls** | **t-test (p-value)** |
| **V1** | | | | 1,2 |
| | | | | |
| Mean | 1611.813 | 1567.75 | 1665.46 | 0.832819 |
| Stdev | 737.8149 | 471.97 | 605.694 | |
| Fold Change | 0.967787 | 0.94133 | | 1, C |
| Group | 1/C | 2/C | | 0.739918 |
| | | | | 2, C |
| | | | | 0.579958 |

| | **Group** | | | |
|---|---|---|---|---|
| **Region** | **1** | **2** | **Controls** | **t-test (p-value)** |
| **NAc** | | | | 1,2 |
| | | | | |
| Mean | 8062.819 | 8644.295 | 7099.24 | 0.642232 |
| Stdev | 2156.662 | 4202.303 | 2639.04 | |
| Fold Change | 1.13573 | 1.217637 | | 1, C |
| Group | 1/C | 2/C | | 0.273168 |
| | | | | 2, C |
| | | | | 0.230739 |

**Table ST9:** Inter-region correlation and age-effect on mtDNA common deletion in DLPFC, STG, V1, and NAc.

| **Correlation** | **CD%-STG** | **CD%-DLPFC** | **CD%-NAc** | **CD %-V1** | **Age** |
|---|---|---|---|---|---|
| **CD%-STG** | 1 | 0.758212 | 0.462624 | 0.821403 | 0.273021 |
| **CD%-DLPFC** | 0.758212 | 1 | 0.531127 | 0.718525 | 0.344293 |
| **CD%-NAc** | 0.462624 | 0.531127 | 1 | 0.369471 | 0.625985 |
| **CD %-V1** | 0.821403 | 0.718525 | 0.369471 | 1 | 0.22768 |
| **Age** | 0.273021 | 0.344293 | 0.625985 | 0.22768 | 1 |
| | | | | | |
| **p-Value** | **CD%-STG** | **CD%-DLPFC** | **CD%-NAc** | **CD %-V1** | **Age** |
| **CD%-STG** | 0 | 3.25E-18 | 0.00106 | 1.11E-22 | 0.008101 |
| **CD%-DLPFC** | 3.25E-18 | 0 | 0.000123 | 3.24E-15 | 0.000833 |
| **CD%-NAc** | 0.00106 | 0.000123 | 0 | 0.010591 | 2.53E-06 |
| **CD %-V1** | 1.11E-22 | 3.24E-15 | 0.010591 | 0 | 0.032892 |
| **Age** | 0.008101 | 0.000833 | 2.53E-06 | 0.032892 | 0 |

**Table ST10:** Inter-region correlation and age-effect on deletions per 10K across DLPFC, STG, V1, and NAc.

| **Correlation** | **Age** | **DLPFC** | **NAc** | **STG** | **V1** |
|---|---|---|---|---|---|
| **Age** | 1 | 0.078035 | -0.00125 | -0.06097 | 0.008678 |
| **DLPFC** | 0.078035 | 1 | -0.01485 | 0.050401 | 0.146984 |
| **NACC** | -0.00125 | -0.01485 | 1 | -0.1166 | -0.29514 |
| **STG** | -0.06097 | 0.050401 | -0.1166 | 1 | 0.00112 |
| **V1** | 0.008678 | 0.146984 | -0.29514 | 0.00112 | 1 |
| | | | | | |
| **p-Value** | **Age** | **DLPFC** | **NACC** | **STG** | **V1** |
| **Age** | 0 | 0.454702 | 0.993438 | 0.563717 | 0.93457 |
| **DLPFC** | 0.454702 | 0 | 0.921972 | 0.633274 | 0.162066 |
| **NAc** | 0.993438 | 0.921972 | 0 | 0.440285 | 0.046454 |
| **STG** | 0.563717 | 0.633274 | 0.440285 | 0 | 0.991544 |
| **V1** | 0.93457 | 0.162066 | **0.046454** | 0.991544 | 0 |

**Table ST11:** Inter-region correlation and age-effect on complex I activity across DLPFC, STG, V1, and NAc.

| Correlation | Complex I activity STG | Complex I Activity DLPFC | Complex I activity V1 | Complex I Activity NAc | Age |
|---|---|---|---|---|---|
| **Complex I activity-STG** | **1.0000** | 0.1768 | 0.0611 | 0.0417 | 0.2694 |
| **Complex I activity-DLPFC** | 0.1768 | **1.0000** | -0.0927 | 0.0328 | 0.1513 |
| **Complex I activity-V1** | 0.0611 | -0.0927 | **1.0000** | 0.1305 | -0.0171 |
| **Complex I activity-NAc** | 0.0417 | 0.0328 | 0.1305 | **1.0000** | -0.1775 |
| **Age** | 0.2694 | 0.1513 | -0.0171 | -0.1775 | **1.0000** |

| P-Value | Complex I activity STG | Complex I Activity DLPFC | Complex I activity V1 | Complex I Activity NAc | Age |
|---|---|---|---|---|---|
| **Complex I activity-STG** | **<.0001** | 0.1122 | 0.5857 | 0.7856 | **0.0144** |
| **Complex I activity-DLPFC** | 0.1122 | **<.0001** | 0.3988 | 0.8306 | 0.1668 |
| **Complex I activity-V1** | 0.5857 | 0.3988 | **<.0001** | 0.3930 | 0.8705 |
| **Complex I activity-NAc** | 0.7856 | 0.8306 | 0.3930 | **<.0001** | 0.2435 |
| **Age** | **0.0144** | 0.1668 | 0.8705 | 0.2435 | **<.0001** |

**Table ST12:** Effect of Medication/Drugs on complex I activity in DLPFC, STG, V1, and NAc using pooled SZ/BD subjects.

| P-value | Complex I activity STG | Complex I Activity DLPFC | Neuroactive Drugs | Complex I activity V1 | Complex I Activity NAc |
|---|---|---|---|---|---|
| **Complex I activity-STG** | **<.0001** | 0.1093 | 0.4905 | 0.3879 | 0.5834 |
| **Complex I activity-DLPFC** | 0.1093 | **<.0001** | **0.0073** | 0.3890 | 0.5848 |
| **Neuroactive Drugs** | 0.4905 | **0.0073** | **<.0001** | 0.1461 | 0.8634 |
| **Complex I activity-V1** | 0.3879 | 0.3890 | 0.1461 | **<.0001** | 0.5101 |
| **Complex I activity-NAc** | 0.5834 | 0.5848 | 0.8634 | 0.5101 | **<.0001** |

**Table ST13:** Effect of Age on number of TOM40, PSD95, and coloc in DLPFC.

| Correlation | Age | PSD95/100µm$^3$ | TOM40/100µm$^3$ | PSD95coloc w/ Tomm40 |
|---|---|---|---|---|
| **Age** | **1.0000** | -0.2159 | -0.0603 | -0.1068 |
| **PSD95/100µm$^3$** | -0.2159 | **1.0000** | **0.4116** | **0.5132** |
| **TOM40/100µm$^3$** | -0.0603 | **0.4116** | **1.0000** | **0.6865** |
| **PSD95coloc w/ Tomm40** | -0.1068 | **0.5132** | **0.6865** | **1.0000** |

| P-Value | Age | PSD95/100µm$^3$ | TOM40/100µm$^3$ | PSD95coloc w/ Tomm40 |
|---|---|---|---|---|
| Age | **<.0001** | 0.2794 | 0.7653 | 0.5959 |
| **PSD95/100µm$^3$** | 0.2794 | **<.0001** | **0.0329** | **0.0062** |
| **TOM40/100µm$^3$** | 0.7653 | **0.0329** | **<.0001** | **<.0001** |
| **PSD95coloc w/ Tomm40** | 0.5959 | **0.0062** | **<.0001** | **<.0001** |

**Table ST14:** Effect of Medication/Drugs on TOM40, PSD95, and Coloc in DLPFC.

| Correlation | PSD95/100µm$^3$ | TOM40/100µm$^3$ | PSD95coloc w/ TOM40 | Neuroactive Drugs |
|---|---|---|---|---|
| **PSD95/100µm$^3$** | **1.0000** | **0.4116** | **0.5132** | -0.3133 |
| **TOM40/100µm$^3$** | **0.4116** | **1.0000** | **0.6865** | -0.1724 |
| **PSD95coloc w/ TOM40** | **0.5132** | **0.6865** | **1.0000** | -0.2477 |
| **Neuroactive Drugs** | -0.3133 | -0.1724 | -0.2477 | **1.0000** |

| p-Value | PSD95/100µm$^3$ | TOM40/100µm$^3$ | PSD95coloc w/ TOM40 | Neuroactive Drugs |
|---|---|---|---|---|
| **PSD95/100µm$^3$** | **<.0001** | **0.0329** | **0.0062** | 0.1116 |
| **TOM40/100µm$^3$** | **0.0329** | **<.0001** | **<.0001** | 0.3898 |
| **PSD95coloc w/ TOM40** | **0.0062** | **<.0001** | **<.0001** | 0.2130 |
| **Neuroactive Drugs** | 0.1116 | 0.3898 | 0.2130 | **<.0001** |

**Figure SF1:** mtDNA copy number correlation across DLPFC, V1, and STG.

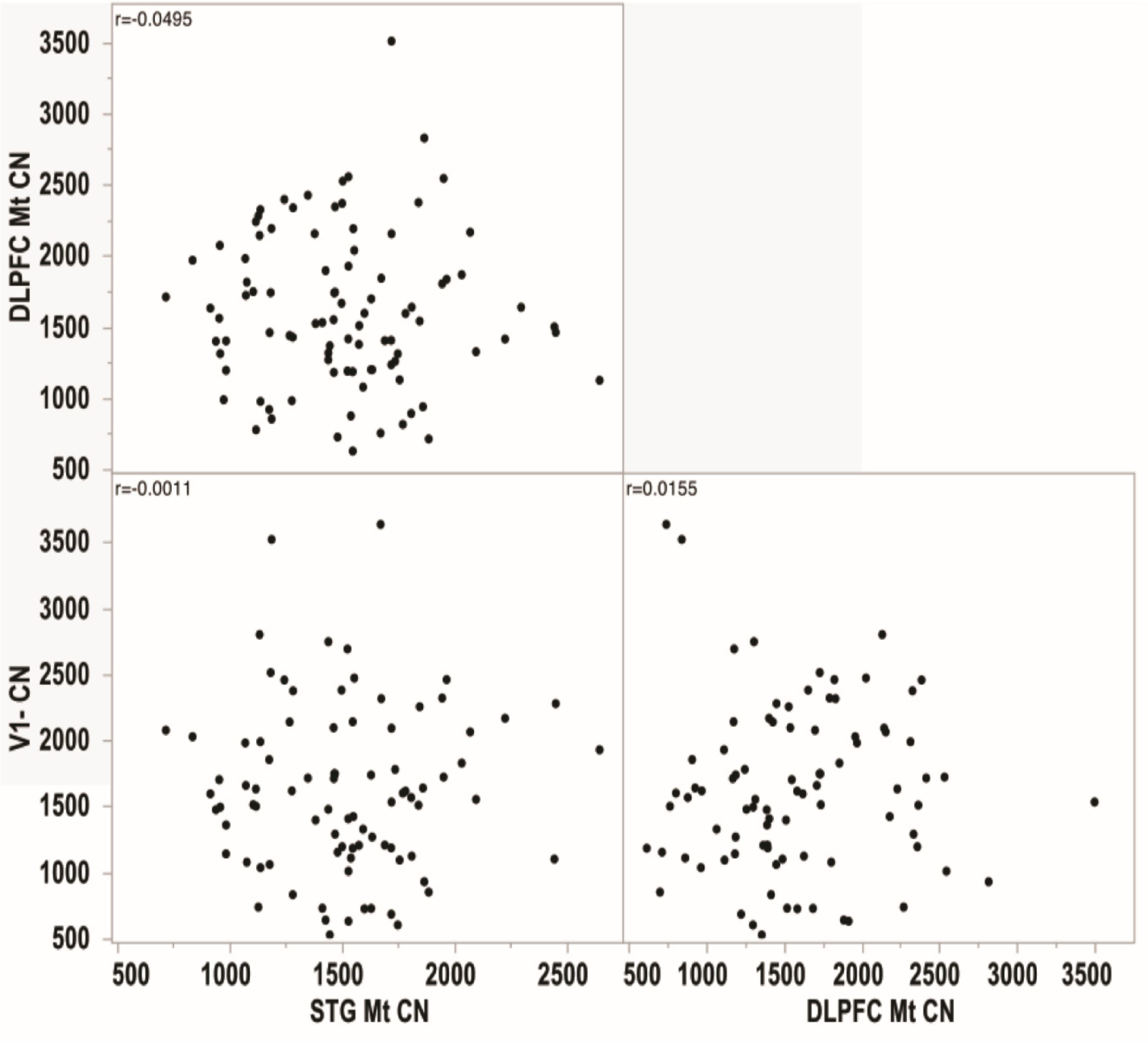

**Figure SF2:** mtDNA common deletion correlation across DLPFC, STG, V1, and NAc.

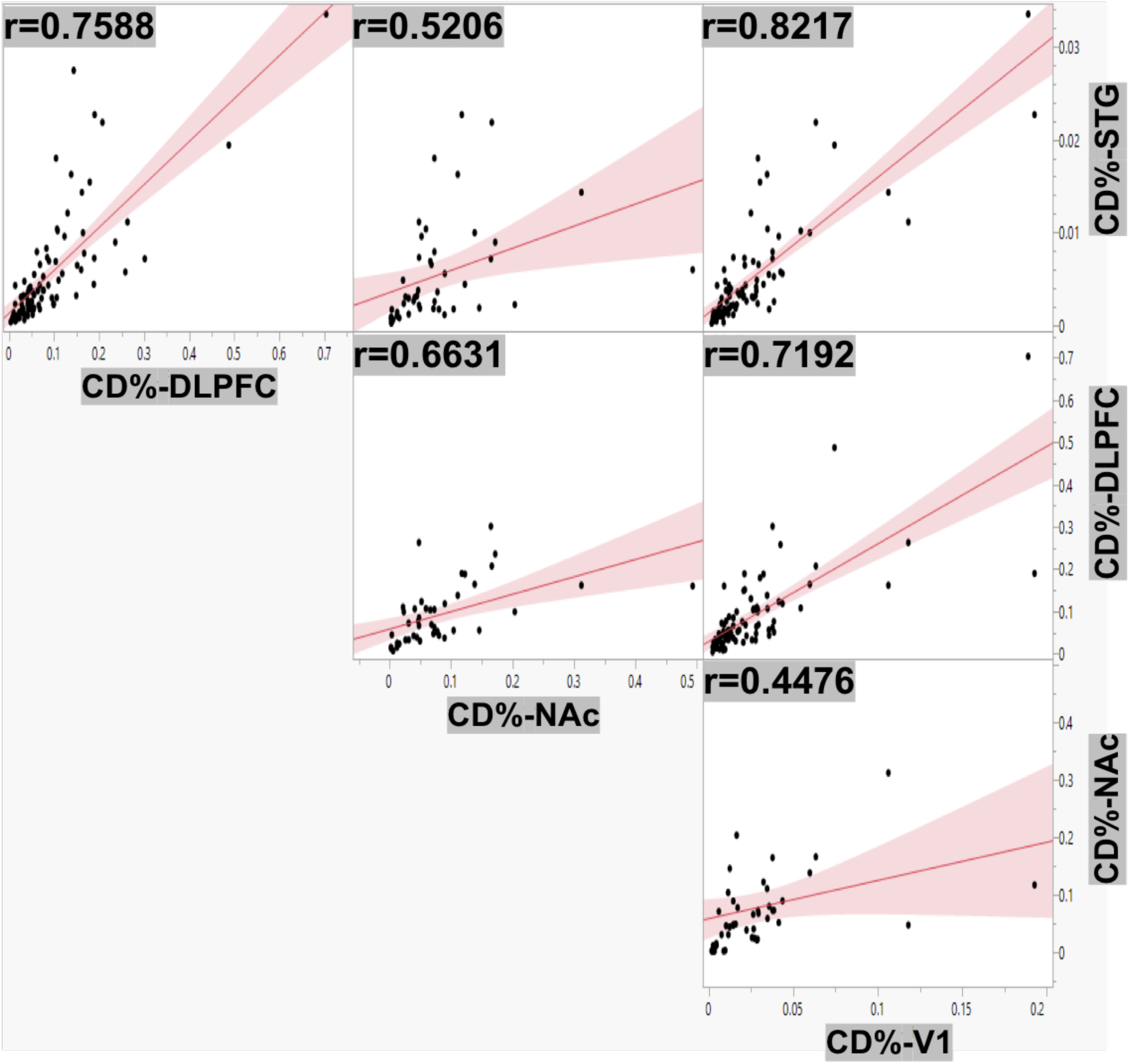

**Figure SF3:** Effect of age on common deletions (%) in DLPFC, STG, V1, and NAc.

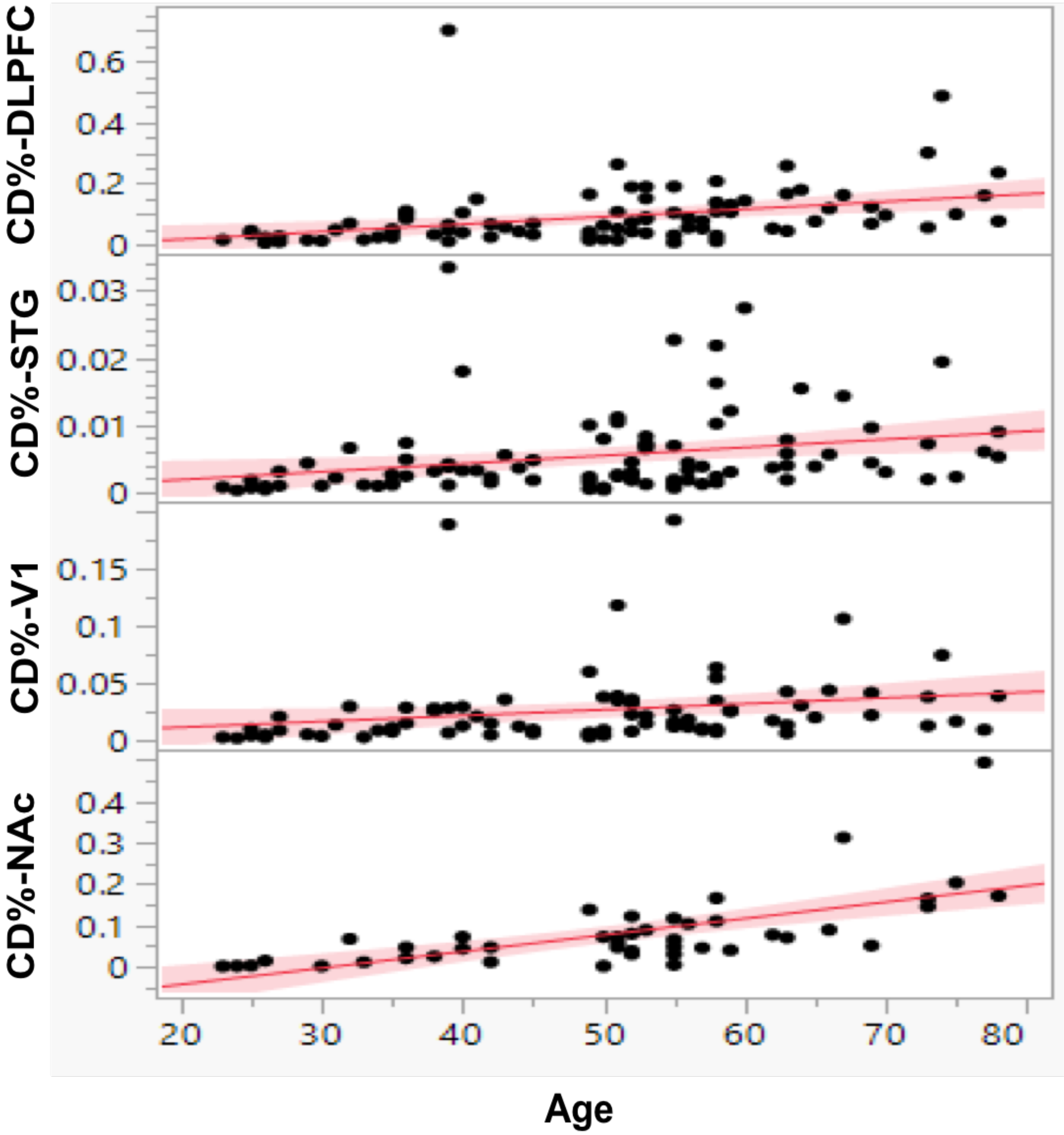

**Figure SF4:** Effect of Medication/Drugs on deletions per 10K in DLPFC, STG, and V1.

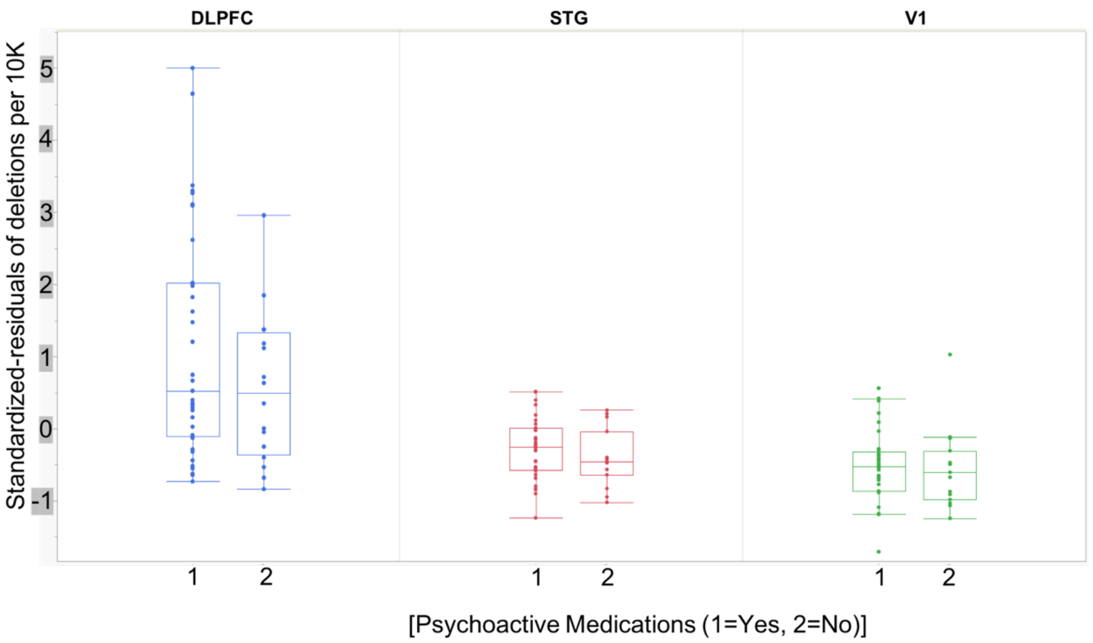


**Figure SF5:** Correlation of standardized residuals of deletions per 10K coverage with complex I activity. The correlation was not statistically significant (p=0.3081) across the three cortical brain regions (DLPFC, STG and V1). Individual brain region correlation were also not significant.

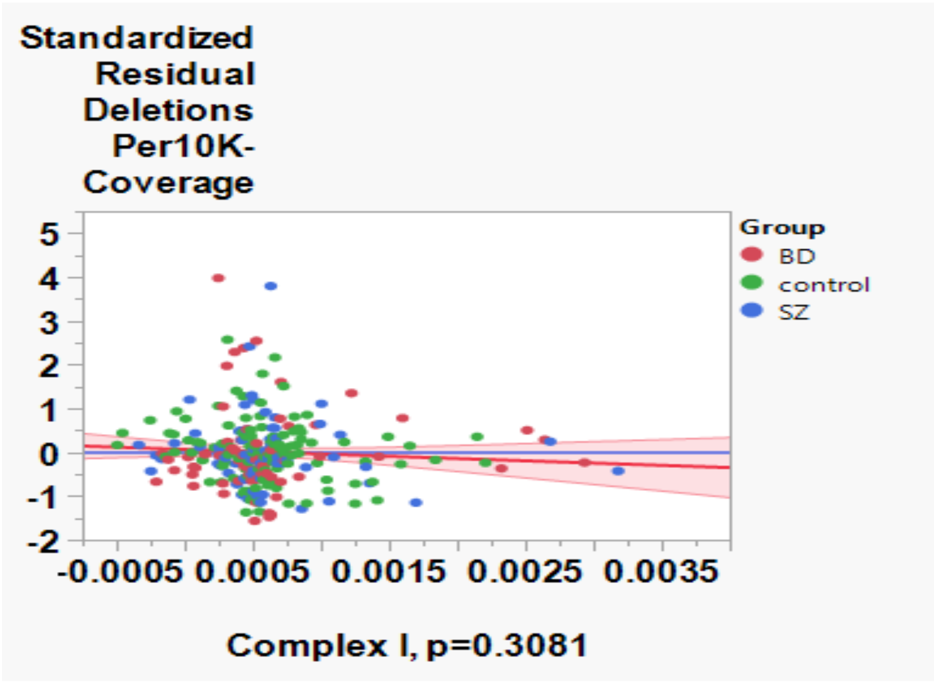

**Figure SF6:** Complex I protein concentration in CON, SZ and BD groups. The samples with low protein concentrations will require higher volumes to obtain equal amounts of proteins. Thus, significantly higher sample volume indicates significantly lower protein concentration. Both SZ and BD groups had significantly lower complex I protein concentration (higher volumes), compared to CON group, across the three cortical brain regions (DLPFC, STG and V1). Data are represented as least square mean (LSM) ± Std. error. p<0.01 is denoted by **, otherwise p>0.05.

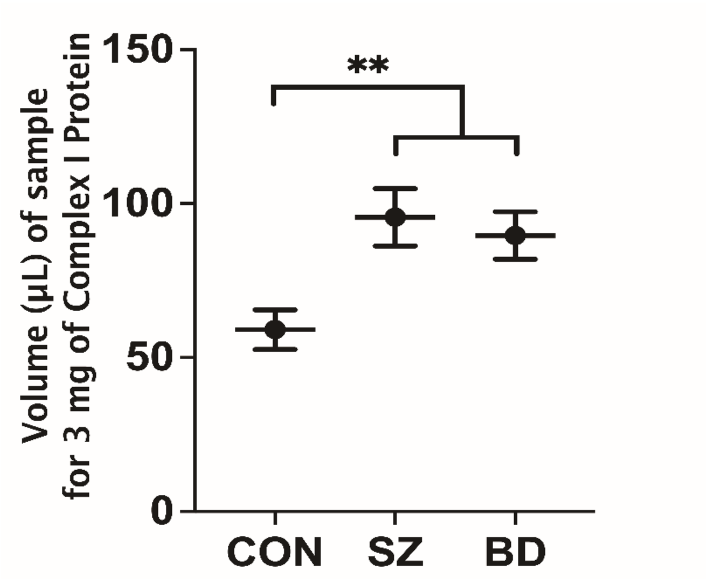


Figure SF7: Role of medications on DLPFC complex I activity. The SZ/BD+Med had significantly lower DLPFC complex I activity compared to CON. The DLPFC complex I activity in SZ/BD-Med groups was not significantly different from CON group. Data are represented as least square mean (LSM) ± Std. error. p<0.01 is denoted by **, otherwise p>0.05.

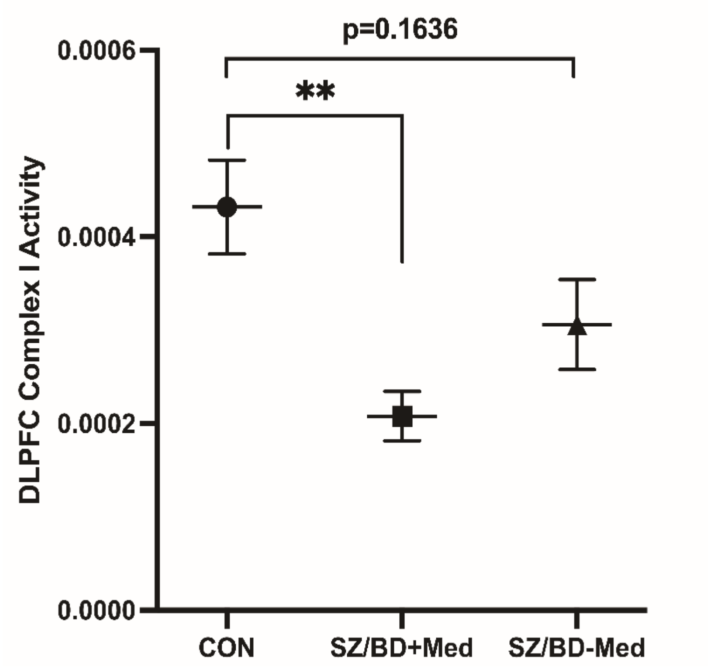


SZ/BD+Med= SZ subjects with medications + BD subjects with medications. (n=41)

SZ/BD-Med= SZ subjects without medications + BD subjects without medications. (n=12)

CON= Control subjects (n=40)

## Supplementary Materials and Methods

### Brain Tissue Dissection:

All the dissections were performed using frozen coronal 1 cm brain sections on dry ice blocks using cooled tools to prevent melting and tissue quality degradation. The PI was not blinded during the experiment, and the co-investigators were not blinded to the group assignment. However, all groups were run simultaneously to avoid batch effect, and to avoid biasing any assay towards a specific group. The primary visual cortex was identified from occipital pole coronal sections using the striate cortex gyrus as a landmark characterized by the presence of the Line of Gennari. The STG was located in frozen sections between the lateral sulcus above and the superior temporal sulcus in the anterior temporal lobe. The NAc was dissected from the ventral portion of the anterior striatum under the internal capsule before the presence of the globus pallidus for consistency. Postmortem subjects went through an extensive review of multiple sources of information including the medical examiner's conclusions, coroner's investigation, medical and psychiatric records, and interviews of the decedents' next-of-kin, in order to rule out subjects with neurological disorders or cancer. Additionally, a neuropathological examination of each brain was conducted in order to exclude cases with cerebrovascular disease (infarcts or hemorrhages), subdural hematoma, brain cancer, or other significant pathological features using high-definition pictures taken every 1 cm before freezing the brain. The subjects were previously diagnosed, and postmortem brain, hence no randomization of treatment was applied. The samples were preselected based upon availability and grant funding. A post hoc power analysis, shows that the experiment with the current sample size and effect size, could be replicated with 70% power to detect significant difference in Complex I in DLPFC between groups. For the colocalization of TOM40 and PSD95, there is a 95% power to replicate the reported effect with the sample size used (n = 47). For Complex I ELISA protein difference in the STG, there is a 62% power to replicate the experiment with the current sample sizes.

### mtDNA Copy Number and Common Deletion:

The total DNA (nDNA+mtDNA) was extracted from DLPFC, STG, V1 and NAc samples using a phenol-chloroform extraction method. The mtDNA CN and mtDNA CD in DLPFC, STG and NAc samples was determined with quantitative real-time PCR (qPCR), whereas mtDNA CN and mtDNA CD in V1 samples were determined using the QX200 Droplet Digital PCR System (Bio-Rad, Hercules, CA, USA). Quantitative real-time PCR was run using the Applied Biosystems 7900HT real-time instrument in 12-µL reaction volumes using 2 ng gDNA and 2 µl of each standard with published conditions in triplicate. For mtDNA CN, a 200-bp gBlock oligonucleotide (Integrated DNA Technologies, Coralville, IA, USA) representing the human albumin gene (ALB) on chromosome 4 is the reference DNA standard. An 11- point standard curve created with this gBlock has copies ranging from 50 - 50,000 copies per µl of the albumin gene. A 326-bp gBlock Gene Fragment (Integrated DNA Technologies, Coralville, IA) for the mitochondrial wild-type sequence (mt13176-13501) is used as a copy number standard for mtDNA. An 11-point standard curve is created with copies ranging from 20,000-10,000,000 per µL of mitochondria wild type product. Mitochondrial DNA CN for the samples is determined using the ratio of mtDNA CN/ALB CN. For the mtDNA CD, a 301-bp gBlock Gene Fragment (Integrated DNA Technologies, Coralville, IA, USA) encompassing the common deletion breakpoints (mt8224–8469 and

mt13447–13501) is used as a standard for the deletion. An 11-point standard curve is created with copies ranging from 50-50,000-copies/µL. CD% is calculated using the following equation: CD% = (mtDNA deletion copies)/ (mtDNA wild type copies + deletion copies) × 100.
The following primers were used:

mtDNA CN/CD based on SYBR Green chemistry:
ALB_Primer_F: 5′-TGCATGAGAAAACGCCAGTA-3′
ALB_Primer_R: 5′-TCTGCATGGAAGGTGAATGT-3′
mtWT_Primer_F: 5′-TTACAATCGGCATCAACCAA-3′
mtWT_Primer_R: 5′-GCTAATGCTAGGCTGCCAAT-3′
mtDel_Primer_F: 5′-AGGGCCCGTATTTACCCTAT-3′
mtDel_Primer_R: 5′-GCTAATGCTAGGCTGCCAAT-3′

mtDNA CN and mtDNA CD in V1 samples were determined using the QX200 Droplet Digital PCR System (Bio-Rad, Hercules, CA, USA). For each assay, a mixture of both the forward and reverse primers and probes were combined. Stock concentrations of each primer and probe were 100 µM, and to make 100 reactions worth of a 20x assay, we used 18 µL of the forward primer stock at 100 µM, 18 µL of reverse primer stock at 100 µM, 5 ul of probe stock at 100 µM and 59 µL of water. The final 1x concentration was 900 nM for each primer and 250 nM for each probe. Each 20 µL reaction consisted of 10 µL 2x ddPCR Supermix (Bio-Rad, Hercules, CA, USA), 1 µL of the above 20x assay consisting of the primers and probe, and the appropriate amount of gDNA and water up to the final 20 µL volume. To run the mitochondria wild-type assay, 0.01 ng of gDNA was used. To run the ALB and common deletion assays 10 ng of gDNA were used. Samples were placed in the QX200 Droplet Generator to partition each sample into 20,000 nanoliter-sized droplets. Droplets were then run on the thermal cycler with the following program: 95 x 10 min, followed by 40 cycles of 94 x 30 sec, then 60 x 1 min. A final enzyme deactivation for 98 x 10 min was followed by a hold at 4. Following PCR amplification, the droplets were analyzed in the QX200 Droplet Reader. QuantaSoft Software (Bio-Rad, Hercules, CA, USA) was used to measure the number of positive and negative droplets for each fluorophore.
The following primers and probe Integrated DNA Technologies, Coralville, IA, USA) were used for ddPCR (all as listed 5’-3’):
Primers for ALB: F-TGCATGAGAAAACGCCAGTA, R- TCTGCATGGAAGGTGAATGT
Probe for ALB: /5HEX/TGGTGAACA/ZEN/GGCGACCATGCT/3IABkFQ/.
For the mito wildtype (WT) assay, the primers and probe were
Primers for WT: F- GGAAGCCTATTCGCAGGATT, R- CTAGGAAAGTGACAGCGAGG
Probe for WT: /56-FAM/CCCCTTCCA/ZEN/AACAACAATCCCC/3IABkFQ/.
For the common deletion (CD) assay the primers and probe were-
Primers for CD: F-AGGGCCCGTATTTACCCTAT, R-GCTAATGCTAGGCTGCCAAT
Probe for CD: /5HEX/CCCCTCTAG/ZEN/AGCCCACTGTAAAGC/3IABkFQ/.

**Pooled Effect of Total Deletion of mtDNA by High-resolution Pipeline "Splice-Break":**

In this manuscript, we present the results of the number of deletion species per 10K coverage as this showed a significant effect across diagnoses. The number of deletions per 10K was calculated by counting the total number of deletion species (a unique set of breakpoints) in a particular

sample, then dividing by the benchmark coverage and multiplying by 10000. The Splice-Break pipeline included long-range (LR) PCR amplification of mtDNA, next-generation sequencing (NGS), and MapSplice (v.2.1.18) algorithm (which detects putative mtDNA deletion breakpoint "junction reads"). Before NGS library preparation, the mtDNA was enriched for each sample using a long-range (LR) PCR utilizing back-to-back primers that hybridize to the control region of the mitochondrial genome. Primer sequences used for most samples were F 5'-CCGCACAAGAGTGCTACTCTCCTC-3' and R 5'-GATATTGATTTCACGGAGGATGGTG-3' (Integrated DNA Technologies). In cases where back-to-back primers failed to amplify the WT mitochondrial genome, the following alternate primers were used: 5'-AAGCCTAAATAGCCCACACG-3' and 5'-GTGGCTTTGGAGTTGCAGTT-3'. For NGS, libraries were prepared using TrueSeq Kit for DLPFC, STG, and V1 samples. Library preparation for NAc samples was performed using the 384 Seq-Well kit (San Diego, CA). NAc Libraries were sequenced as 150-mer paired-end reads on a patterned flow cell using the Illumina NovaSeq6000 at the UCI Genomics High Throughput Facility. DLPFC, STG, and V1 libraries were sequenced as 150-mer paired-end reads on Illumina HiSeq2500 at the UCI Genomics High Throughput Facility. We used our custom bash script Splice-Break.sh (https://github.com/brookehjelm/Splice-Break/) to perform automated filtering, normalization, and annotation of mtDNA deletion breakpoints and performed manual calculations of the cumulative deletion metrics.

**Complex I Activity (activity/unit of complex I protein):**

Briefly, brain tissue (60–100 mg) was homogenized in PBS at 4°C and centrifuged at 3000 rpm for 5 min. After centrifugation, the first supernatant is collected and measured for protein concentration to ensure adequate protein for both ELISA and activity assays. Equivalent amounts of supernatant protein are extracted (Part 8201086, Abcam, Cambridge, UK), the second supernatants are collected (16000xg, 4°C, 20 min) and stored at –80°C for complex I ELISA and activity assays. The complex I ELISA assay (AB178011, Abcam, Cambridge, UK) quantitatively measures NADH dehydrogenase protein. Equal protein amounts are used with a specific monoclonal capture and detection antibodies reactive to CI isolates. All standards, samples, and controls are run in duplicate in 96-well plates pre-coated with a capture antibody provided in the Abcam kit. A standard curve prepared from the HeLa lyophilized cell extract and additional standards prepared from a postmortem human PFC brain extract are used. The standard CI ELISA protein curve is calculated from the regression of the average OD 450 nm reading of each HeLa standard against their concentrations. The second part of the assay is the CI activity assay. Each brain sample is diluted to the same CI protein (amount determined above by ELISA) to determine the rate of CI activity. All samples and controls were run in duplicate according to the manufacturer's protocol (AB109721, Abcam, Cambridge, UK). The activity rate is expressed as the change in absorbance per minute using this equation: complex I activity for each sample (milli-optical density units, mOD/min) = (1,000 × ΔOD450/30 min) where Δ represents the final minus initial OD450.

**Immunohistochemistry (IHC) for Colocalization of Mitochondria and Synaptic Marker:**

Fresh frozen DLPFC tissue blocks (~1 $cm^3$) were immersion-fixed in freshly made 4% PFA (in PBS) for 24 hours at 4°C. Post-fixation, tissue blocks were cryopreserved in 20% sucrose and 0.1% Na-Azide in PBS for 3 days, then placed in 30% sucrose for 3 days at 4°C. After cryopreservation, blocks are stored frozen at -80°C until cryo-sectioning. Free-floating 10 µm tissue cryostat sections are collected in 0.1% sodium azide in PBS. Antigen-retrieval was performed by incubating free-floating tissue sections in 1 mM EDTA buffer (pH 8) at 100°C and high pressure for 5 min. After antigen retrieval, slices were incubated in PBS-Triton (0.3%) for 1 hour. Slices were blocked with 10% goat serum. Primary antibodies for excitatory postsynaptic markers (PSD95) (MA1-045, Thermofisher) and mitochondria (TOM40) (ab185543, Abcam) were added and incubated for 24 hours at 4°C. The next day, slices were washed with PBS three times, followed by respective secondary antibodies incubation for 24 hours at 4°C. After washing secondary antibodies, slices were incubated with DAPI as a nuclear counterstain for 10 min. Next, slices were incubated in 70% ethanol for 5 min followed by incubation with an auto-fluorescence eliminator reagent (Sigma Aldrich, Cat#2160) for 1 min. Slides were mounted in Prolong diamond antifade solution (Thermofisher, P36970). Digital z-stack images are collected using a Zeiss LSM 780 confocal microscope with oil X 63 objective (NA=1.4). Acquired image parameters: voxel size 0.07 x 0.07 x 0.32 $micron^3$, 1024 x 1024 pixels with a depth of 12 bit. Colocalization analysis was performed through IMARIS software (Bitplane). The spots of TOM40 and PSD95 with a diameter of greater than 1.2 µm were excluded from the analysis. The colocalization was defined as the number of TOM40 spots within the distance of 0.25 µm from PSD95 spots. 5-8 images from 2-3 sections per subject were captured. The total number of subjects evaluated for IHC was 51.
